# Integrating large language models in biostatistical workflows for clinical and translational research

**DOI:** 10.1017/cts.2025.10064

**Published:** 2025-05-30

**Authors:** Steven C. Grambow, Manisha Desai, Kevin P. Weinfurt, Christopher J. Lindsell, Michael J. Pencina, Lacey Rende, Gina-Maria Pomann

**Affiliations:** 1 Department of Biostatistics and Bioinformatics, Duke University School of Medicine, Durham, NC, USA; 2 Department of Medicine, Stanford University School of Medicine, Stanford, CA, USA; 3 Department of Population Health Sciences, Duke University School of Medicine, Durham, NC, USA

**Keywords:** Biostatistics, large language models, collaborative research, artificial intelligence, clinical research

## Abstract

**Introduction:**

Biostatisticians increasingly use large language models (LLMs) to enhance efficiency, yet practical guidance on responsible integration is limited. This study explores current LLM usage, challenges, and training needs to support biostatisticians.

**Methods:**

A cross-sectional survey was conducted across three biostatistics units at two academic medical centers. The survey assessed LLM usage across three key professional activities: communication and leadership, clinical and domain knowledge, and quantitative expertise. Responses were analyzed using descriptive statistics, while free-text responses underwent thematic analysis.

**Results:**

Of 208 eligible biostatisticians (162 staff and 46 faculty), 69 (33.2%) responded. Among them, 44 (63.8%) reported using LLMs; of the 43 who answered the frequency question, 20 (46.5%) used them daily and 16 (37.2%) weekly. LLMs improved productivity in coding, writing, and literature review; however, 29 of 41 respondents (70.7%) reported significant errors, including incorrect code, statistical misinterpretations, and hallucinated functions. Key verification strategies included expertise, external validation, debugging, and manual inspection. Among 58 respondents providing training feedback, 44 (75.9%) requested case studies, 40 (69.0%) sought interactive tutorials, and 37 (63.8%) desired structured training.

**Conclusions:**

LLM usage is notable among respondents at two academic medical centers, though response patterns likely reflect early adopters. While LLMs enhance productivity, challenges like errors and reliability concerns highlight the need for verification strategies and systematic validation. The strong interest in training underscores the need for structured guidance. As an initial step, we propose eight core principles for responsible LLM integration, offering a preliminary framework for structured usage, validation, and ethical considerations.

## Introduction

Biostatisticians, informaticists, and other data scientists are at the forefront of leveraging advanced computational tools in scientific research. These scientists collaborate closely with clinical domain specialists to design studies, manage and house data, perform data analysis, present results, and interpret findings. Their work involves understanding the clinical problem, navigating complicated analytical and data generation processes, conducting literature reviews, writing and debugging code, interpreting results, and translating statistical concepts for non-technical collaborators.

Large language models (LLMs) are advanced artificial intelligence (AI) systems trained on vast amounts of text data, capable of understanding and generating human-like text that can help data scientists transform their approach to scientific research and collaboration. Data scientists are uniquely positioned to capitalize on emerging LLM technologies to overcome numerous challenges, including the demand for their skill sets in an increasingly data-intensive research environment. Integrating LLMs into data science workflows offers innovative solutions to address these challenges and augment human expertise. Dell’Acqua et al. [1] and Peng et al. [2] demonstrated that these tools can streamline research processes, enhance productivity, and allow experts to focus on higher-level analytical tasks. However, their integration is complex, requiring careful navigation of multiple challenges: potential errors and hallucinations that necessitate robust verification strategies, ethical considerations around data privacy and bias mitigation, and ensuring transparency while balancing AI capabilities with human expertise [3].

Recent publications have explored LLM applications in clinical research and biostatistics, highlighting their potential to enhance research workflows. These studies highlight LLMs’ capabilities in generating scientific text, guiding clinical trial design, optimizing code, synthesizing medical literature, and facilitating communication of technical findings [4–7]. Guidelines and practical strategies have been proposed to ensure ethical implementation and maintain scientific rigor [8–10]. However, specialty-specific guidance is still needed for integrating these tools into workflows.

To address this need, we conducted a cross-sectional survey in September and October 2024 among three biostatistics units at Duke University School of Medicine and Stanford University School of Medicine to assess LLM usage, barriers to use, and training needs. This timeframe represents a specific moment in LLM development and adoption, when consumer-grade LLMs were becoming available to biostatisticians but before the widespread integration of these tools into operating systems and development environments. While the survey focused on biostatisticians, its insights apply broadly to data scientists in healthcare research. By analyzing patterns of LLM usage, practical applications, challenges, and training needs, we propose a set of key principles to provide guidance for effectively incorporating LLMs into biostatistical practice.

## Methods

### Core competency framework

This study used a framework established by Pomann et al. [11] and recently validated by Slade et al. [12], defining three core domains of biostatistical practice in collaborative research settings. The first domain, *communication and leadership*, encompasses how biostatisticians communicate complex quantitative methodology to interdisciplinary collaborators. This requires strong written and verbal communication skills, effective meeting strategies, project management, and assertive advocacy of statistical perspectives, described as “strong statistical voice.”

The second domain, *clinical and domain knowledge*, reflects how biostatisticians understand research contexts, including study designs, clinical measures, and disease mechanisms. This knowledge enables critical evaluation of whether existing methods are suitable or new approaches are needed.

The third domain, *quantitative expertise*, forms the cornerstone of biostatistical practice. It encompasses core statistical and computing skills for applying rigorous methodologies in collaborative research settings. This domain directly impacts the quality and validity of research results, enabling biostatisticians to develop complex statistical models, interpret data accurately, and provide reliable insights that inform clinical and scientific decisions.

### Study design

The study used a cross-sectional survey to evaluate LLM usage in biostatistical workflows. Data collection utilized the Qualtrics [13] platform for survey administration.

### Participants and survey administration

Participants were recruited via email from three academic biostatistics units: the Duke Clinical Research Institute (DCRI) Biostatistics group, the Duke Biostatistics, Epidemiology, and Research Design (BERD) Methods Core, and Stanford University’s Quantitative Sciences Unit (QSU). The eligible population included 208 biostatisticians (162 staff, 46 faculty).

The survey was structured to assess LLM use across the three core competency domains. Initial questions gathered demographics and organizational context. The main survey sections then examined (1) communication and leadership tasks (e.g., writing, presenting, and explaining statistical concepts), (2) clinical and domain knowledge activities (e.g., understanding medical terminology, reviewing methods), and (3) quantitative expertise applications (e.g., coding, statistical analysis, methodology).

Respondents answered questions about each domain’s specific LLM applications, perceived usefulness, errors encountered, and verification strategies. The survey employed branching logic to direct non-users of LLMs to specific questions about barriers and training needs (see *Survey Instrument and Flow Diagram Report* in the supplementary materials). The Duke University Health System Institutional Review Board determined that the study met the criteria for exemption from further IRB oversight (Pro00116592).

### Data analysis

Quantitative analyses consisted primarily of frequency tables with counts and percentages, performed in R [14] via RStudio [15] with systematic data cleaning and denominator adjustments for survey skip patterns. We report the three most frequent responses in the main text for multiple-choice questions with numerous response options, with complete frequency distributions available in the *Detailed Survey Analysis Report* in the supplementary materials.

Throughout the broader research process, we utilized several LLMs for different tasks: ChatGPT-4o [16], Claude 3.5 Sonnet [17], and Microsoft Copilot [18] for writing, coding, and documentation, and ChatGPT O1 preview [19] for resolving complex analytical challenges. All LLM-generated content underwent thorough human review. Results were compiled, and the manuscript was prepared using Quarto [20] to enhance computational reproducibility.

We combined human expertise with ChatGPT-4o and Claude 3.5 Sonnet to qualitatively analyze free-text responses. The process began with parallel independent coding by a human analyst and the LLMs to identify themes, followed by systematic integration and reconciliation. Free-text responses underwent systematic preprocessing for word frequency analysis using R’s tidytext package to provide quantitative validation of identified themes. For questions about effective prompts, where respondents could provide up to three examples, we analyzed the responses as a combined corpus to identify cross-cutting themes in how biostatisticians formulate LLM queries.

## Results

### Survey participant characteristics and LLM usage

Among 208 eligible biostatisticians (162 staff, 46 faculty) across three academic units, 69 (33.2%) responded to the survey. Of these, 68 reported their institutional affiliation: 29 (42.6%) were affiliated with DCRI Biostatistics, 25 (36.8%) with Stanford QSU, and 14 (20.6%) with Duke BERD. Among the 69 respondents, 56 (81.2%) held staff positions, and 57 (82.6%) identified as biostatisticians.

More than half of respondents (44/69, 63.8%) reported using LLMs in their work. Among those who answered the frequency question (43/69), 20 (46.5%) reported using them daily and 16 (37.2%) weekly. OpenAI ChatGPT (33/44, 75.0%) and Microsoft Copilot (22/44, 50.0%) were the most widely used tools, with many users employing multiple LLMs for different tasks. Institutional access to Microsoft Copilot varies by version and licensing model. While both institutions provide access to Copilot, full Microsoft 365 Copilot is not universally deployed and typically requires additional licensing by individual users or departments. ChatGPT and Copilot remained the primary tools across all competency domains, with ChatGPT usage ranging from 60 to 62% and Copilot usage from 26 to 38% depending on the domain. While 34 out of 69 (49.3%) reported receiving encouragement to use LLMs, fewer had access to formal guidelines (13, 18.8%) or training (13, 18.8%). See Table 1 for complete participant and LLM usage characteristics.

**Table 1.** Participant demographics and large language model (LLM) Usage characteristics

Characteristic	n	%
***Total respondents***	***69***	
**Institution**	**68**	
DCRI Biostatistics	29	42.6%
Stanford QSU	25	36.8%
Duke BERD	14	20.6%
**Professional role**	**69**	
Staff	56	81.2%
Faculty	12	17.4%
Other	1	1.4%
**Experience level**	**68**	
0–2 years	18	26.5%
3–5 years	14	20.6%
6–10 years	16	23.5%
11+ years	20	29.4%
**LLM usage**	**69**	
Yes	44	63.8%
No	25	36.2%
**LLM usage frequency**	**43**	
Daily	20	46.5%
Weekly	16	37.2%
Monthly or less	7	16.3%
LLM tools used* ^**‡**^	**44**	
OpenAI ChatGPT	33	75.0%
Microsoft Copilot (formerly Bing Chat)	22	50.0%
Google Gemini	9	20.5%
Hugging Face LLMs	4	9.1%
Anthropic Claude	3	6.8%
GitHub Copilot	3	6.8%
Meta LLaMA	3	6.8%
Other (Perplexity)	1	2.3%
**Organizational support** ^**†**^	**69**	
Encouragement to use LLMs	34	49.3%
Guidance or guidelines on appropriate LLM use	13	18.8%
Training on how to use LLMs	13	18.8%
None of the above	9	13.0%

LLM = large language model; DCRI = Duke Clinical Research Institute; QSU = Quantitative Sciences Unit; BERD = Biostatistics, Epidemiology, and Research Design; *n* = number of respondents; % = percentage of respondents. * Multiple-select question; percentages may exceed 100%. ^†^ Organizational support question was implemented as single-select though intended as multiple-select. ^‡^ Institutional access to listed LLM tools varies. Some tools, such as Microsoft Copilot, may be available through enterprise agreements, but full feature access may require additional individual or departmental licensing.

### Survey results by core competency domain

#### Communication and leadership

Among the 38 users who employed LLMs for communication tasks, 29 (76.3%) used them for “*editing and improving writing quality*,” 27 (71.1%) for “*composing emails or other messages*,” and 18 (47.4%) for “*explaining statistical concepts to non-experts*.” Most respondents found LLMs valuable for these tasks, with 23 (62.2%) rating them “*very useful*” and 14 (37.8%) “*somewhat useful*” out of 37 total respondents.

Respondents described specific applications, with one participant noting they “*generally use LLMs to do grammar check (e.g., emails, SAP, report, manuscript) or revise a paragraph that I wrote*.” Another highlighted using LLMs for formal writing tasks, requesting to “*Create the project summary for this NIH proposal using the attached research strategy using no more than 30 lines of text*.”

#### Clinical and domain knowledge

Among the 35 users who employed LLMs for clinical and domain knowledge tasks, 24 (68.6%) used them for self-learning through “*defining or explaining medical terms and concepts*,” 14 (40.0%) for “*understanding and interpreting clinical measurement scales (e.g., PROMIS, PHQ-9)*,” and 12 (34.3%) for “*summarizing scientific papers or reports*.” Respondents found value in these tasks, with 15 (44.1%) rating them “*very useful*” and 19 (55.9%) “*somewhat useful*” out of 34 total respondents.

Respondents described specific strategies through both their experiences and example prompts. One shared their experience: “*I used to Google the new clinical concepts when I started to work on a project before LLMs came out, and now LLMs are good at providing definitions.*” Another provided an effective prompt they use: “*You are a knowledgeable assistant in clinical research. Explain in simple terms the difference between Type 1 and Type 2 diabetes, focusing on the physiological mechanisms and treatment implications.*” This mix of direct experience and example prompts showcases different approaches biostatisticians use to leverage LLMs for understanding clinical concepts.

#### Quantitative expertise

Among the 40 users who employed LLMs for quantitative expertise tasks, 31 (77.5%) used them for “*writing, debugging, or documenting code (e.g., R, Python, SAS)*,” 23 (57.5%) for “*explaining statistical methods or concepts*,” and 21 (52.5%) for “*learning new statistical techniques or software*.” Respondents found value in these tasks, with 17 (44.7%) rating them “*very useful*” and 21 (55.3%) “*somewhat useful*” out of 38 total respondents.

Respondents shared various effective prompts related to quantitative expertise. One participant described their detailed approach: “*I’ll ask for code assistance in statistical modeling tasks suitable for NIH grants or medical articles. Tasks include data preparation, exploratory analysis, and model development. The code should be clear, follow best practices, and include explanations. Ensure variable recoding is verified with head(), tail(), and table() for accuracy. Your code should always include comments explaining what the code is doing. You should assume that variables you may be asked to recode could include missing values. Your code should always check to see if needed packages are already loaded and install them if necessary.*” Another participant shared a template they use when requesting SAS procedure examples: “*Give an example of using SAS proc XXX to do _____________. Start with dataset “begin,” with the variable “A” representing _______, “B” representing __________, and “C” representing _________.*” The underscores represent placeholders where the user fills in specific procedure names, analysis goals, and variable definitions. These responses showcase practical approaches biostatisticians use to leverage LLMs for coding and analysis tasks.

### Challenges and training needs

Beyond LLM usage patterns, our survey explored usage challenges and training needs. Among 41 users who responded to questions about encountering errors, 29 (70.7%) encountered incorrect answers that, if unidentified, could have had significant consequences, while 12 (29.3%) reported no significant errors. For those who provided detailed descriptions, the thematic analysis revealed four primary categories of errors:

1. **Incorrect Code Generation**: LLMs generated non-existent functions or incorrectly mixed existing ones. One participant noted: *“Chat GPT has written incorrect R code… It invents functions that don’t exist and mixes functions that do exist.”*
2. **Statistical Misinterpretation**: LLMs misinterpreted statistical results. For example: *“…while interpreting the odds ratio, it treated exposure as outcome and vice versa. That changes the meaning…*”
3. **Content Fabrication**: LLMs generated fictional content, with one respondent reporting: *“…added content to meeting minutes that was not discussed*.”
4. **Inappropriate Style or Tone**: Communication issues were common, particularly in professional correspondence: *“Copilot does not do a good job at drafting emails. It’s tone is robotic…*”

These categories of errors highlight distinct challenges in incorporating LLMs into professional workflows. Errors in code generation and statistical misinterpretation pose particular risks in analytical tasks, where subtle mistakes could propagate undetected through analyses. Content fabrication threatens document integrity, particularly in formal research communications, while inconsistent tone and phrasing may undermine professional interactions.

Recent systematic reviews highlight notable improvements in LLMs’ capabilities for code generation, model sophistication, and reliability [21]. Developments such as GitHub Copilot [22], ERNIE-Code [23], and autonomous AI software engineers like Devin [24] illustrate the expanding role of LLMs and broader AI systems in software development. Yet, as our survey results indicate, users continue to report challenges applying these tools, despite ongoing advancements.

Among respondents who described how they identified errors that could have led to significant consequences, four primary approaches emerged:

1. **Expertise and Prior Knowledge**: Respondents relied on existing knowledge: *“I know the content that I teach and I know when the LLM is wrong.”*
2. **External Verification**: Users cross-referenced trusted sources: *“If I am unsure of an answer… I usually do a web search as well or check some sources to try and see if it aligns with what the LLM gave me.”*
3. **Testing and Debugging**: For code, respondents tested output systematically: *“I carefully checked output on my test data and noticed anomalies… based on those anomalies, I went back to the code, and found and fixed the problem quickly.”*
4. **Manual Review**: Careful inspection helped catch LLM-generated errors: *“When I was preparing how I was going to explain it to the collaborator and reading it over, I realized the variable names were wrong and some of the key steps were incorrect and some were missing.”*

These verification approaches varied by task. Quantitative analyses emphasized systematic testing and debugging, while clinical knowledge required external verification of terminology and methodology. Communication tasks benefited from manual review and peer feedback, particularly for tone and accuracy.

The organizational context surrounding these challenges reveals an important gap: while nearly half of the respondents received encouragement to use LLMs, few had access to formal training or verification protocols. This lack of structured support aligns with the strong interest in additional training resources. Among the 58 respondents who answered this question, the most frequently requested resources were “*case studies or best practice examples of LLM use in my field*” (44, 75.9%), “*interactive tutorials or guided practice with real-world examples of using LLMs*” (40, 69.0%), and “*structured training sessions or courses (workshops, seminars, webinars, online courses)*” (37, 63.8%).

Among the 25 non-LLM users who responded, key barriers included “*I haven’t had the time to learn how to use them effectively*” (20, 80.0%), “*I have concerns about the accuracy or reliability of the outputs*” (11, 44.0%), and “*I don’t believe they would be useful for the specific tasks I do in my workflow*” (9, 36.0%). The most commonly cited factors that would encourage them to use LLMs included “*more training or resources specifically tailored to my field*” (20, 80.0%), “*successful case studies or examples from my colleagues*” (14, 56.0%), and “*more evidence of their accuracy and reliability*” (12, 48.0%).

In the final survey question, respondents were asked if they had any other thoughts or observations about using LLMs in biostatistical workflows. Five key themes emerged:

1. **Productivity Enhancement**: Respondents noted efficiency gains: *“I think it is very helpful for coding and things I would normally web search/look on stack overflow for I can just ask the LLM and get code.”*
2. **Reliability Concerns**: Users highlighted accuracy issues, particularly with specialized tasks: *“One thing I noticed is that ChatGPT is not always accurate when pulling references or providing citations. Sometimes, the references it provides do not exist in the real world…”*
3. **Need for Critical Oversight**: Respondents emphasized verification importance: *“Please discourage others from sharing LLM-derived results until they have confirmed the accuracy… If I want to ask chatGPT I will do so myself. If I am asking a human chat group then I want a human response.”*
4. **Domain-Specific Limitations**: Users noted constraints in specialized applications: *“For biostatistical/methodological topics, LLMs tend to be fairly shallow. However, they are good for summaries, and can often be a good starting place…”*
5. **Future Potential**: Despite limitations, some saw opportunities ahead: *“I feel like a first draft of a SAP might be able to be written by an LLM… If we can figure out how to do that it would save a great deal of time…”* Others suggested that LLMs *“represent a huge potential productivity boost”* while emphasizing the importance of careful verification.

These reflections reinforce the mixed perspectives on LLMs – while they offer efficiency gains and new opportunities, concerns about reliability, domain limitations, and the need for robust verification persist. To support responsible integration, we propose a set of key principles for incorporating LLMs into biostatistical workflows, offering a structured approach to usage, validation, and ethical considerations.

### Key principles for responsible LLM use in biostatistical workflows

**Figure 1.**
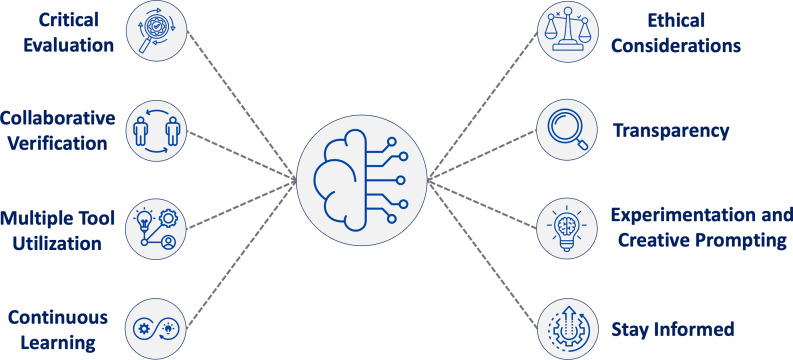
Eight guiding principles for responsible large language model (LLM) use in biostatistical workflows. These principles were developed as a synthesis of findings from our survey – particularly reported usage barriers, verification strategies, and ethical concerns – alongside a review of responsible AI literature and our professional experience as early adopters in academic biostatistics. This framework is not a direct representation of survey frequencies but is instead intended to offer early guidance on best practices. Issues such as federal grant policy restrictions and risks of intellectual property exposure through public application programming interfaces (APIs) fall within the scope of principles such as ethical considerations, transparency, and multiple tool integration.

In addition to workforce training for LLM use, clear processes for implementing best practices with these tools are needed. Based on our survey analysis, literature review, and professional experience using LLMs, we propose eight core principles for effective LLM use in biostatistical workflows (Figure 1). These principles, grounded in respondents’ experiences with verification strategies, usage barriers, and general observations, represent an initial framework for responsible LLM usage in biostatistical practice – one that will evolve with continued use and technological advancement.

These principles reflect key challenges and opportunities identified by survey respondents and draw from broader discussions on responsible LLM practices and verification strategies:

1. **Critical Evaluation:** Use personal judgment, domain expertise, and trusted sources to independently assess LLM outputs before integrating them into biostatistical work.
2. **Collaborative Verification:** Engage colleagues or supervisors to review LLM outputs, offering complementary perspectives that help identify errors or misinterpretations.
3. **Multiple Tool Integration:** Combine resources and approaches to ensure comprehensive verification.
4. **Continuous Learning:** Use LLMs to augment existing biostatistical knowledge while ensuring users possess the domain expertise necessary to critically evaluate and verify outputs.
5. **Ethical Considerations:** Ensure alignment with institutional guidelines and regulatory requirements, particularly regarding data privacy, security, and bias. Where possible, consider using enterprise-grade systems that safeguard confidential or sensitive information.
6. **Transparency:** Maintain clear documentation of LLM use and verification processes.
7. **Experimentation and Creative Prompting:** Embrace creative experimentation in LLM interactions, recognizing that different approaches to framing prompts can yield significantly different results. This includes using LLMs to draft potential approaches or propose code, as well as to reflect on or refine work already in progress.
8. **Stay Informed:** Given rapid technological advancement, regularly monitor LLM developments and emerging best practices, particularly new models optimized for code generation.

## Discussion

Among 208 eligible biostatisticians at two major academic medical centers, our survey achieved a 33.2% response rate (69/208). More than half of respondents (44, 63.8%) reported using LLMs in their work; of the 43 who answered the frequency question, 20 (46.5%) used them daily and 16 (37.2%) weekly. Given the typical lag between study design, data analysis, and publication, our findings likely reflect AI practices that will shape clinical and translational research studies appearing in the literature over the next several years. Moreover, understanding current biostatistical workflows provides an important context for interpreting clinical and translational research publications emerging today, many of which reflect practices from the same period captured in our survey.

LLM use was highest for “*writing, debugging, or documenting code (e.g., R, Python, SAS)*” (31/40, 77.5%), followed by “*editing and improving writing quality*” (29/38, 76.3%) and “*composing emails or other messages*” (27/38, 71.1%). Most respondents found LLMs valuable for their tasks, with the highest perceived usefulness in communication (23/37, 62.2% rated “very useful”) and quantitative expertise (17/38, 44.7% rated “very useful”), while 15/34 (44.1%) rated clinical and domain knowledge applications as “very useful.”

As organizations formalize policies, training, and infrastructure around LLMs, broader adoption may extend beyond early users. Institutional agreements likely influence tool selection, as evidenced by the high prevalence of Microsoft Copilot usage among respondents. This suggests that adoption is influenced not only by user preference but also by enterprise partnerships, licensing structures, and ongoing institutional caution and integration.

Recent studies of LLM adoption in technical workflows parallel our findings while highlighting biostatistics-specific considerations. While data scientists follow similar interaction patterns, Chopra et al. [25] found that practitioners spend significant time on prompt preparation and validation, reflecting the careful verification approaches our participants described. However, our study reveals additional complexity in biostatistical applications, where concerns extend beyond code correctness to statistical validity and clinical implications.

Verification challenges appear consistently across the three professional domains assessed in our survey – communication, clinical knowledge, and quantitative expertise. Low et al. [26] found that general-purpose LLMs produced relevant, evidence-based answers in only 2%–10% of clinical applications, while domain-specialized systems achieved 24%–58% – supporting our participants’ emphasis on domain-specific adaptation. Similarly, our survey found that 70.7% of respondents encountered errors that could have led to significant consequences, reinforcing the need for rigorous verification strategies. These findings highlight a broader issue in data-intensive fields, including biostatistics, where errors can propagate through analytical workflows if not properly validated. While emerging techniques such as content-grounded prompting and agentic LLM workflows show promise in addressing these challenges, they were not widely available or implemented during our study. Our analysis reflects usage patterns during the early retail-access phase when most users interacted with general-purpose tools without dedicated infrastructure for grounding or orchestration.

Building on these observations, prior research has further highlighted the importance of domain-specific validation, particularly in biostatistics and regulated environments where incorrect outputs can pose significant risks. Denecke et al. [27] emphasized that LLM validation should be domain-specific. Komandur et al. [28] found substantial variation in LLM performance when analyzing biomedical data, demonstrating that outputs shift based on prompting strategies and model architecture. Similarly, Lee et al. [29] highlighted inconsistencies in LLM evaluation methodologies across healthcare applications, emphasizing the lack of standardized validation frameworks and the challenge of ensuring reproducibility.

Several key limitations contextualize our findings. The relatively low response rate of 33.2% (69/208 eligible respondents) raises concerns about response bias, as individuals already using or interested in LLMs may have been more likely to participate in the survey. This is particularly relevant given that our recruitment targeted biostatisticians within our institutions. Additionally, our sample from two academic medical centers with established biostatistics groups may not represent experiences across all biostatistical practice settings. Thus, our findings may reflect early adopters rather than typical usage patterns, limiting generalizability.

Importantly, even when LLM tools are widely accessible, integration into daily biostatistical practice may lag substantially. This underscores the need for researchers to clearly document how and when LLMs are incorporated into scientific workflows, as practices vary across settings and over time. Nevertheless, this focus on early adopters provides valuable insights into emerging practices and implementation challenges that will become increasingly relevant as adoption broadens across the biostatistical community.

Our reliance on self-reported interactions rather than direct analysis of LLM use presents another limitation. While we collected examples of effective prompts, we did not systematically analyze how different LLM models varied in their responses to the same statistical queries. This limitation becomes increasingly significant as LLM technology evolves – during the study period and manuscript preparation (September 2024–February 2025), major advancements occurred across all leading companies, introducing new functionalities, model updates, and shifts in behavior. Notable releases included OpenAI’s GPT-4o, GPT-4o with Canvas, o1, and o3; Anthropic’s Claude 3.5 Sonnet and Claude Artifacts; Google’s Gemini 2.0; and DeepSeek’s open-source breakthroughs [30]. These developments introduced improved image processing, direct computer interaction, enhanced project management capabilities, and significantly improved code generation, further altering the landscape of LLM capabilities. As Bedi et al. [31] noted, many LLM evaluations focus on accuracy without addressing real-world variability, model drift, or contextual reasoning – factors that may influence reliability over time.

Future research should explore how model updates impact biostatistical workflows, particularly regarding the consistency of statistical interpretations. Since LLM performance is based on prompt design, computational constraints, and evolving training data, developing standardized, reproducible validation frameworks will be critical to ensuring long-term reliability in biostatistical applications. Additionally, our exclusive focus on text-based LLMs rather than broader generative AI represents another scope limitation. While multimodal applications such as image generation and audio content creation will likely become relevant to biostatistical workflows over time, they were not widely adopted by biostatisticians during our study period. Future investigations should track the integration of these expanded capabilities as the generative AI ecosystem evolves. Addressing these challenges will require structured investigations into LLM verification methods and long-term model consistency. These limitations, combined with our findings, suggest three important research priorities.

First, longitudinal and experimental evaluation studies should assess how LLM capabilities and adoption patterns evolve, incorporating objective productivity and work quality measures, such as time to task completion, error rates, satisfaction, and first-pass code accuracy, rather than relying solely on self-reported data. Research should examine individual and team-based usage patterns, particularly in relation to innovative organizational models such as dedicated prompt engineering specialists serving as internal consultants. These specialists could guide best practices, assist with prompt engineering, and support verification efforts within biostatistics teams.

Second, building on these findings and prior work demonstrating variable performance in biomedical contexts, the development and evaluation of domain-specific implementations should be a research priority. Future studies should investigate various approaches to domain adaptation in biostatistical workflows, including customized LLM interfaces, dedicated workspace environments, and knowledge-augmented architectures that can securely incorporate domain-specific content. These efforts may also benefit from emerging techniques such as content-grounded prompting and agentic architectures, which could help improve reliability and reduce hallucination by anchoring outputs in curated knowledge sources or enabling cross-verification among systems. Emerging AI agents designed to orchestrate full analytic pipelines may further enhance efficiency, although their success will depend heavily on access to high-quality, domain-specific training data and transparent orchestration frameworks.

Comparative studies evaluating these approaches could provide valuable insights into their relative effectiveness, security, and practicality. Given the substantial error rate reported by respondents, specialized verification protocols and domain-adapted solutions may offer clear advantages over general-purpose LLMs for biostatistical applications.

Third, organizational implementation strategies require systematic investigation. Our findings show limited formal support: while nearly half of respondents received encouragement to use LLMs, fewer than twenty percent had access to formal guidelines or training. Expanding training initiatives, particularly for biostatisticians without prior experience with LLMs, could further support responsible adoption. In addition to training and access, institutions may consider secure infrastructure, such as enterprise LLM environments, to protect sensitive data better and support safe implementation. Research into the effectiveness of different training models (e.g., peer mentoring, interactive tutorials, or embedded support teams) is needed to guide best practices. A consensus-building approach, such as a Delphi process involving diverse stakeholders, could refine and validate these preliminary principles as the technology and implementation patterns mature. Future research should also explore ethical implications, including bias mitigation, transparency in model outputs, and reproducibility standards to ensure that LLM-generated content aligns with rigorous scientific principles.

Given these challenges, structured guidance is needed to ensure responsible LLM integration in biostatistics. Our findings reinforce the need for clear principles that support methodological integrity, scientific rigor, and ethical responsibility. Drawing on our survey results, literature review, and professional experience, we propose eight core principles to guide biostatisticians in engaging LLMs effectively and responsibly. While not yet formally validated, these principles represent an important starting point for shaping practice. Future efforts should explore how these guidelines can be operationalized in institutional settings and refined as LLM technologies continue to evolve.

## Conclusion

This survey offers early insights into LLM usage among biostatisticians in academic medical centers, highlighting productivity benefits and integration challenges. Although LLMs enhance efficiency, especially for coding, writing, and communication, their responsible use demands rigorous verification and systematic validation. Respondents expressed strong interest in educational resources, underscoring the need for structured training, formal guidelines, and institutional support.

As LLM technologies continue to evolve, standardized verification frameworks and domain-specific adaptations will be essential for maintaining statistical rigor. Our proposed core principles offer a foundational framework for structuring LLM use in biostatistical practice. Future research should focus on refining these principles, developing domain-specific adaptations, and implementing institutional strategies for training, governance, and ethical oversight, ensuring transparent, reproducible, and scientifically sound LLM-assisted workflows.

## Supporting information

10.1017/cts.2025.10064.sm001 Grambow et al. supplementary material Grambow et al. supplementary material

## Supplementary material

For supplementary material/s referred to in this article, please visit https://doi.org/10.1017/cts.2025.10064
