# Supplementary material for: Integrating large language models in biostatistical workflows for clinical and translational research
Source: J Clin Transl Sci. 2025 May 30;9(1):e131. doi: 10.1017/cts.2025.10064 (PMC12260977; doi:10.1017/cts.2025.10064)
Supplement: Grambow et al. supplementary material [file S2059866125100642sup001.pdf]

Supplementary Materials

Supplementary Materials

Integrating Large Language Models in Biostatistical Workflows for Clinical and Translational Research

Contents

Appendix 1: Survey Instrument and Flow Diagram
- Full survey instrument, including all questions and response options
- Survey structure and flow diagram

Appendix 2: Detailed Survey Analysis Report
- Summary statistics for all survey questions
- Thematic analysis of free-text responses
- Word frequency analyses
- Cross-tabulations and data visualizations

Appendix 1: Survey Instrument and Flow Diagram

Table of contents

1 Introduction **2**

2 Q1. Welcome and Consent **3**

3 Demographics and Context **4**

3.1 Q2. Name *(Optional)* 4
3.2 Q3. Email Address *(Optional)* 5
3.3 Q4. Professional Role 5
3.4 Q5. Professional Title 5
3.5 Q6. Years of Experience 5
3.6 Q7. Institutional Affiliation 5
3.7 Q8. Organizational Support 6

4 LLM Usage Assessment **6**

4.1 Q9. Current LLM Usage 6

5 LLM User Path **6**

5.1 Q10. Usage Frequency 6
5.2 Q11. LLMs Used 6
5.3 Communication and Leadership Tasks 7
5.3.1 Q12. Communication Tasks Used 7
5.3.2 Q13. Communication Tasks Usefulness 7
5.3.3 Q14. Preferred LLM for Communication 7
5.4 Clinical and Domain Knowledge Tasks 8
5.4.1 Q15. Clinical Knowledge Tasks Used 8
5.4.2 Q16. Clinical Knowledge Tasks Usefulness 8
5.4.3 Q17. Preferred LLM for Clinical Knowledge 8

5.5 Quantitative Expertise Tasks . . . 9
5.5.1 Q18. Quantitative Tasks Used . . . 9
5.5.2 Q19. Quantitative Tasks Usefulness . . . 9
5.5.3 Q20. Preferred LLM for Quantitative Tasks . . . 10
5.6 Experience with Errors . . . 10
5.6.1 Q21. Error Encounters . . . 10
5.6.2 Q22. Error Description . . . 10
5.6.3 Q23. Error Recognition . . . 10
5.7 Effective Prompts . . . 10
5.7.1 Q24. Prompt Collection . . . 10
5.7.2 Q25. First Prompt . . . 11
5.7.3 Q26. Second Prompt . . . 11
5.7.4 Q27. Third Prompt . . . 11

6 Non-LLM User Questions **11**
6.1 Q28. Reasons for Non-Use . . . 11
6.2 Q29. Incentives for Future Use . . . 11

7 Common Questions (All Respondents) **12**
7.1 Q30. Better Utilize LLMs . . . 12
7.2 Q31. Additional Thoughts . . . 12

1 Introduction

This document presents the complete survey instrument used in “Integrating Large Language Models in Biostatistical Workflows for Clinical and Translational Research,” a cross-sectional study conducted from September to October 2024 at Duke and Stanford Universities. The survey was designed to assess how biostatisticians and related professionals integrate Large Language Models (LLMs) into their workflows across three core competency domains: communication and leadership, clinical and domain knowledge, and quantitative expertise.

The survey comprised 30 questions (Q2-Q31) organized into four sections:

1. Demographics and organizational context
2. LLM usage patterns and frequency
3. Specific applications across competency domains
4. Implementation challenges and training needs

To efficiently capture different user experiences, the instrument employed branching logic that

directed respondents down separate paths based on their LLM usage status. The flow diagram below provides a visual representation of this structure, with each box corresponding to specific question groups. Following the diagram, we present the complete survey instrument with all questions, response options, and branching logic preserved exactly as implemented through the Qualtrics platform.

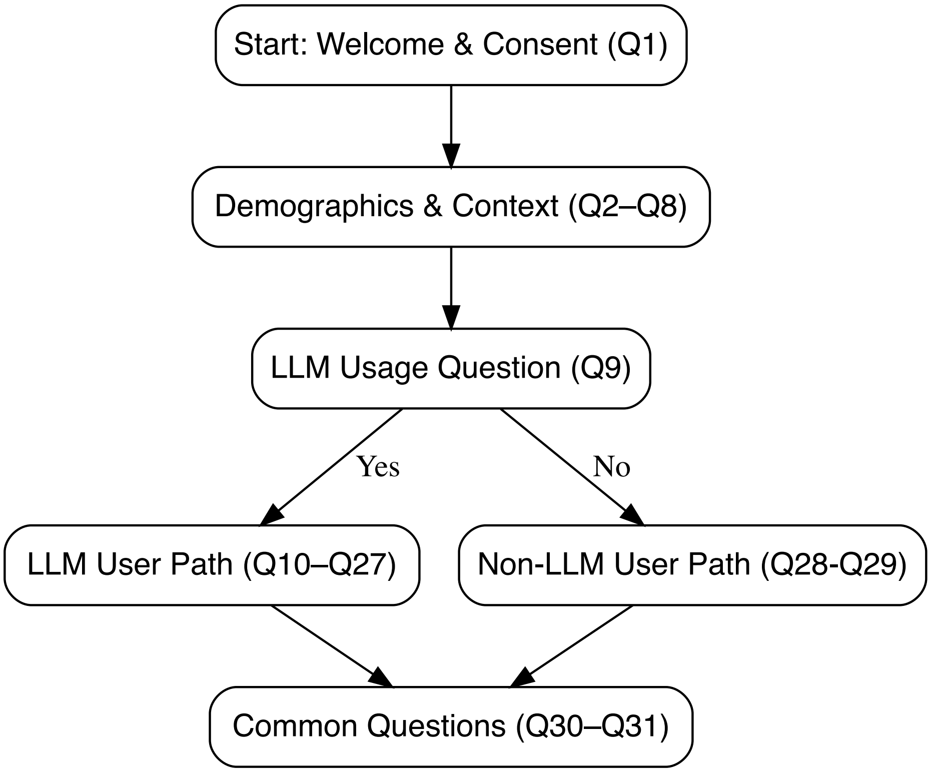

Figure 1: Survey Flow Diagram

2 Q1. Welcome and Consent

Welcome to the survey on using Large Language Models (LLMs) in biostatistical workflows. Thank you for participating. Your participation is invaluable in helping us understand how professionals like you utilize LLMs (e.g., OpenAI ChatGPT, Anthropic Claude, Meta Llama, Google Gemini, Microsoft Copilot, GitHub Copilot) in your daily work.

This survey focuses on three key areas:

Communication and Leadership: How LLMs assist in explaining concepts, writing reports, or interacting with colleagues.

Clinical and Domain Knowledge: How LLMs help in understanding clinical concepts, study designs, data, regulations, and staying updated on new developments.

Quantitative Expertise: How LLMs aid statistical analysis, data interpretation, and coding tasks.

Your responses are important to us and will be handled with care. We will not collect any personally identifiable information, such as your name or email address, unless you choose to provide it. If you do provide your name and/or email address, it will only be used to contact you for potential follow-up questions or to clarify your responses, and it will not be linked to any of your survey responses in any publications. Your anonymized responses, including any prompts or examples you share, may be used in research publications to illustrate key findings. All data will be used solely for research purposes to improve our understanding of LLM integration in biostatistical practice.

Key Participant Information

Your participation in this survey is voluntary.

Completing this survey implies your consent to participate in this research study.

You may choose to provide your name and/or email address, but this is optional.

All responses, including any prompts or examples, will be anonymized and used only for research.

The results of this survey, including selected anonymized quotes or examples, may be published in a manuscript on this topic.

Please answer each question as honestly and accurately as possible. You will be directed to a separate set of questions if you are not currently using LLMs.

If you have any questions or concerns, please contact Lacey Rende at [email address].

The Institutional Review Board (IRB) is a group of people who review research to protect your rights. If you have a question about your rights as a participant, would like to discuss problems or concerns, have questions or want to offer input, or want to obtain additional information, you should contact the Duke University Health System Institutional Review Board (IRB) Office at (919) 668-5111.

Thank you for your time and valuable contribution!

3 Demographics and Context

3.1 Q2. Name *(Optional)*

[Text entry field]

3.2 Q3. Email Address *(Optional)*

[Text entry field]

3.3 Q4. Professional Role

Which of the following best describes your work role?

- Staff
- Faculty
- Other (please specify) [Text entry field]

3.4 Q5. Professional Title

Which of the following best describes your professional title?

- Biostatistician
- Bioinformatician
- Data Scientist
- Informaticist
- Statistical Programmer
- Other (please specify) [Text entry field]

3.5 Q6. Years of Experience

How many years of experience do you have in your current role?

- 0-2 years
- 3-5 years
- 6-10 years
- 11+ years

3.6 Q7. Institutional Affiliation

Which institution and group are you currently affiliated with?

- Duke BERD
- DCRI Biostatistics
- Stanford QSU

3.7 Q8. Organizational Support

Has your managing organization provided any of the following regarding the use of LLMs (e.g., OpenAI ChatGPT, Anthropic Claude, Meta Llama, Google Gemini, Microsoft Copilot, GitHub Copilot)?

(Note: This question was implemented as single select in Qualtrics but was intended to be multiple select. Results should be interpreted accordingly.)

- Encouragement to use LLMs
- Guidance or guidelines on appropriate LLM use
- Training on how to use LLMs
- None of the above

4 LLM Usage Assessment

4.1 Q9. Current LLM Usage

Are you currently using LLMs in your work?

- Yes [Continue to LLM User Questions]
- No [Skip to Non-LLM User Questions]

5 LLM User Path

5.1 Q10. Usage Frequency

How often do you use LLMs in your work?

- Daily
- Weekly
- Monthly
- Rarely

5.2 Q11. LLMs Used

Which LLMs have you used in your work? (Select all that apply)

- OpenAI ChatGPT
- Google Gemini

- Anthropic Claude
- Meta LLaMA
- Microsoft Copilot (formerly Bing Chat)
- GitHub Copilot
- xAI Grok
- Hugging Face LLMs
- Other (please specify) [Text entry field]

5.3 Communication and Leadership Tasks

5.3.1 Q12. Communication Tasks Used

Do you use LLMs for any of the following communication and leadership tasks in your work? (Check all that apply)

- Drafting meeting agendas or notes
- Composing emails or other messages
- Writing sections of grant proposals or manuscripts
- Drafting recommendation letters or letters of support
- Generating content for presentation slides
- Editing and improving writing quality
- Explaining statistical concepts to non-experts
- Brainstorming ideas or outlining projects
- Summarizing long documents or conversations
- Learning about organizational structures at my institution
- Other (please specify) [Text entry field]
- I do not use LLMs for communication and leadership tasks [Skip to Q15]

5.3.2 Q13. Communication Tasks Usefulness

How useful are LLMs for **communication and leadership** tasks in your work?

- Very useful
- Somewhat useful
- Not at all useful

5.3.3 Q14. Preferred LLM for Communication

Which LLM do you use the most for **communication and leadership** tasks?

- OpenAI ChatGPT
- Google Gemini
- Anthropic Claude
- Meta LLaMA
- Microsoft Copilot (formerly Bing Chat)
- GitHub Copilot
- xAI Grok
- Hugging Face LLMs
- Other (please specify) [Text entry field]

5.4 Clinical and Domain Knowledge Tasks

5.4.1 Q15. Clinical Knowledge Tasks Used

Do you use LLMs for any of the following **clinical and domain knowledge** tasks in your work? (Select all that apply)

- Summarizing scientific papers or reports
- Identifying relevant research papers or studies
- Defining or explaining medical terms and concepts
- Critically evaluating research methods in papers, grants, or protocols
- Understanding and interpreting clinical measurement scales (e.g., PROMIS, PHQ-9)
- Selecting appropriate outcome measures for specific research questions or studies
- Staying informed about new developments in your field
- Interpreting and utilizing clinical guidelines or best practice
- Understanding and interpreting data security guidelines or IRB requirements
- Other (please specify) [Text entry field]
- I do not use LLMs for clinical and domain knowledge tasks [Skip to Q18]

5.4.2 Q16. Clinical Knowledge Tasks Usefulness

How useful are LLMs for **clinical and domain knowledge** tasks in your work?

- Very useful
- Somewhat useful
- Not at all useful

5.4.3 Q17. Preferred LLM for Clinical Knowledge

Which LLM do you use the most for **clinical and domain knowledge** tasks?

- OpenAI ChatGPT
- Google Gemini
- Anthropic Claude
- Meta LLaMA
- Microsoft Copilot (formerly Bing Chat)
- GitHub Copilot
- xAI Grok
- Hugging Face LLMs
- Other (please specify) [Text entry field]

5.5 Quantitative Expertise Tasks

5.5.1 Q18. Quantitative Tasks Used

Do you use LLMs for any of the following **quantitative expertise** tasks in your work (Select all that apply)

- Creating or reviewing statistical analysis plans (SAPs)
- Writing, debugging, or documenting code (e.g., R, Python, SAS)
- Explaining statistical methods or concepts
- Interpreting the results of statistical analyses
- Learning new statistical techniques or software
- Generating synthetic data or simulating datasets
- Extracting or transforming data from various sources (e.g., PDFs, websites)
- Performing calculations or data transformations (e.g., generating derived variables, re-coding variables, creating summary statistics)
- Automating repetitive data cleaning or analysis tasks
- Other (please specify) [Text entry field]
- I do not use LLMs for quantitative expertise tasks [Skip to Q21]

5.5.2 Q19. Quantitative Tasks Usefulness

How useful do you find LLMs for **quantitative expertise** tasks in your work?

- Very useful
- Somewhat useful
- Not at all useful

5.5.3 Q20. Preferred LLM for Quantitative Tasks

Which LLM do you use the most for **quantitative expertise** tasks?

- OpenAI ChatGPT
- Google Gemini
- Anthropic Claude
- Meta LLaMA
- Microsoft Copilot (formerly Bing Chat)
- GitHub Copilot
- xAI Grok
- Hugging Face LLMs
- Other (please specify) [Text entry field]

5.6 Experience with Errors

5.6.1 Q21. Error Encounters

Have you encountered instances where an LLM provided an incorrect answer that could have had significant negative consequences if not recognized?

- Yes
- No [Skip to Q24]

5.6.2 Q22. Error Description

Please describe one such situation and the potential impact it could have had on your work if it had gone unnoticed: [Text entry field]

5.6.3 Q23. Error Recognition

How did you recognize that the LLM's answer was incorrect in this instance? [Text entry field]

5.7 Effective Prompts

5.7.1 Q24. Prompt Collection

Thinking of your use of LLMs for the three key areas we have focused on (**communication and leadership**, **clinical and domain Knowledge**, and **quantitative expertise**), please share up to three prompts that you have found particularly effective in your work:

5.7.2 Q25. First Prompt

Effective prompt 1: [Text entry field]

5.7.3 Q26. Second Prompt

Effective prompt 2: [Text entry field]

5.7.4 Q27. Third Prompt

Effective prompt 3: [Text entry field]

6 Non-LLM User Questions

6.1 Q28. Reasons for Non-Use

Which of the following best describes your reasons for not using LLMs in your work? (Select all that apply)

- I haven't had the time to learn how to use them effectively.
- I have concerns about the accuracy or reliability of the outputs.
- I have ethical concerns about the use of LLMs in my field.
- I lack access to adequate training or resources on how to use them.
- I don't believe they would be useful for the specific tasks I do in my workflow.
- I have concerns about data privacy or security when using LLMs.
- My organization has restrictions or policies against using LLMs.
- Other (please specify) [Text entry field]

6.2 Q29. Incentives for Future Use

What would make you more likely to use LLMs in your work? (Select all that apply)

- More evidence of their accuracy and reliability
- Easier-to-use interfaces or platforms
- More training or resources specifically tailored to my field
- Clearer guidelines or ethical frameworks for LLM use
- Improved data privacy and security features
- Successful case studies or examples from my colleagues
- Access to a work-approved LLM platform or tool
- Other (please specify) [Text entry field]

7 Common Questions (All Respondents)

7.1 Q30. Better Utilize LLMs

What type of training or support would help you better utilize LLMs in your work? (Select all that apply)

- Structured training sessions or courses (workshops, seminars, webinars, online courses)
- Interactive tutorials or guided practice with real-world examples of using LLMs
- Access to online forums or discussion groups focused on LLMs
- Mentoring or coaching from experienced LLM users
- Comprehensive documentation and user guides for LLMs
- Case studies or best practice examples of LLM use in my field
- Other (please specify) [Text entry field]

7.2 Q31. Additional Thoughts

Do you have any other thoughts or observations about the use of LLMs in biostatistical workflows that you'd like to share? [Text entry field]

Appendix 2: Detailed Survey Analysis Report

Table of contents

1 **Executive Summary** **2**
1.1 Study Design and Data Collection 3
1.2 Data Cleaning and Preprocessing 3
1.3 Analytical Methodology 3
1.3.1 Quantitative Analysis 3
1.3.2 Qualitative Analysis Framework 4
1.3.3 Technical Implementation 4
1.4 Participant Categorization and Response Classification 5
1.5 Summary of Respondent Categories 5
1.6 Survey Responders 5

2 **Participant Demographics and Organizational Context** **6**
2.1 Professional Role (Q4 & Q5) 6
2.2 Experience Level (Q6) 7
2.3 Institutional Affiliation (Q7) 8
2.4 Organizational Support for LLMs (Q8) 9

3 **LLM Usage - Key Split** **9**
3.1 LLM Usage Status (Q9): Overview of LLM Users vs. Non-Users 9
3.2 Usage Frequency (Q10): Summary of Frequency of LLM Use 10

4 **Analysis by LLM Users** **10**
4.1 LLM Tools Used (Q11) 11
4.2 LLM Usage Categories 11
4.2.1 Communication and Leadership Tasks (Q12–Q14) 11
4.2.2 Clinical and Domain Knowledge (Q15–Q17) 12
4.2.3 Quantitative Expertise Tasks (Q18–Q20) 14

4.3 Experience with Incorrect LLM Answers (Q21) 15
4.4 Analysis of LLM Mistakes (Q22) 16
4.4.1 Findings from Manual Thematic Analysis 16
4.5 Thematic Analysis of How Mistakes Were Recognized (Q23) 19
4.5.1 Findings from Manual Thematic Analysis 19
4.6 Thematic Analysis of Effective Prompts (Q24-Q27) 22
4.6.1 Findings from Manual Thematic Analysis 22

5 Analysis by Non-LLM Users 25
5.1 Reasons for Not Using LLMs (Q28) 26
5.2 Incentives for Future Use (Q29) 27

6 Common Questions (Asked to All Respondents) 27
6.1 Training Needs for LLMs (Q30) 28
6.2 Analysis of General Observations on LLM Use (Q31) 29
6.2.1 Findings from Manual Thematic Analysis 29

1 Executive Summary

This report presents detailed analysis results from a survey examining how Large Language Models (LLMs) are integrated into biostatistical workflows within clinical and translational research. It supplements the main manuscript, "Integrating Large Language Models in Biostatistical Workflows for Clinical and Translational Research," by providing expanded statistical results, methodological detail, and additional data not included in the primary paper.

Grounded in the competency framework developed by Pomann et al. (2021) [1] and validated by Slade et al. (2023) [2], the analysis explores LLM usage across three key domains: Communication and Leadership, Clinical and Domain Knowledge, and Quantitative Expertise. This appendix includes:

- Frequency distributions for all survey questions
- Thematic synthesis of free-text responses
- Word frequency visualizations
- Comprehensive cross-tabulations

Although raw free-text responses were collected, they are not included in this report to protect participant anonymity. Instead, a structured thematic analysis summarizes key insights.

These findings provide the analytical foundation for the conclusions presented in the main manuscript and offer additional context for readers seeking a deeper exploration of LLM-

related practices in biostatistics.

1.1 Study Design and Data Collection

This cross-sectional survey was conducted from September to October 2024 among biostatisticians at Duke and Stanford Universities. It was designed to assess how LLMs are being integrated into biostatistical workflows across three core competency domains: communication and leadership, clinical and domain knowledge, and quantitative expertise.

Administered through the Qualtrics platform [3], the instrument included 31 items (Q1–Q31), with 30 substantive questions (Q2–Q31) organized into four sections:

1. Demographics and organizational context
2. LLM usage patterns and frequency
3. Specific applications across competency domains
4. Implementation challenges and training needs

Branching logic directed respondents to distinct paths based on their LLM usage status. The Duke University Health System Institutional Review Board determined that the study met the criteria for exemption from further IRB oversight (Pro00116592).

1.2 Data Cleaning and Preprocessing

Data preparation followed several key steps:

- Included only responses submitted on or after the official survey launch date (September 20, 2024
- Removed survey previews with no submitted responses
- Retained partial responses for analysis
- Classified respondents as “Completers,” “Partial Responders,” or “Non-Respondents”
- Removed optional names and email addresses after initial counts
- Ensured that no personally identifiable information was used in LLM-assisted analysis

1.3 Analytical Methodology

1.3.1 Quantitative Analysis

Quantitative analysis was conducted in R, using frequency tables with counts and percentages. Denominator adjustments were made to account for branching logic and partial completions.

1.3.2 Qualitative Analysis Framework

We applied a structured thematic analysis to free-text responses, combining human review with LLM-assisted synthesis to identify key patterns. To protect confidentiality, raw free-text responses are not included in this report. Instead, we present a thematic synthesis summarizing the core insights.

1. **Human–LLM Collaborative Thematic Analysis**
   - Independent parallel coding by a human analyst and LLMs (ChatGPT 4o [4], Claude 3.5 Sonnet [5])
   - Integration and reconciliation of identified themes
   - Systematic comparison and team validation of final themes
2. **Computational Text Analysis**
   - Preprocessing using the `tidytext` package in R
   - Word frequency analysis and visualizations (e.g., word clouds, bar plots)
3. **Synthesis and Validation**
   - Cross-method triangulation of findings
   - Validation against original text
   - LLMs used to assist with theme articulation and summary generation

While word frequency analysis offered a broad view of commonly used terms, structured thematic analysis provided deeper interpretive value. This framework was consistently applied across all free-text items, including those related to error descriptions, verification strategies, and general LLM usage observations.

1.3.3 Technical Implementation

The analysis was implemented in R [6] using RStudio [7] and Quarto [8] for computational reproducibility. Key features included:

1. **Modular Workflow**
   - Encapsulated functions for different question type
   - Version-controlled, date-stamped `.R`, `.rds`, and `.RData` files
   - Automated report generation via Quarto
   - Manual version tracking through filename conventions
2. **LLM Integration**
   - Collaborative use of multiple LLMs:
     - ChatGPT 4o
     - Claude 3.5 Sonnet
     - Microsoft Copilot [9]

- ChatGPT O1 Preview [10]

- Human review of all LLM-assisted content
- Iterative refinement of analysis pipelines

1.4 Participant Categorization and Response Classification

The survey was distributed to 208 eligible biostatisticians (162 staff and 46 faculty) across three academic units. A total of **69** individuals submitted usable responses and were included in the analysis, representing **33.2**% of the eligible population.

Respondents were categorized into three groups:

- **Non-Respondents** – individuals who viewed the survey but did not submit any responses
- **Partial Responders** – those who answered some but not all applicable questions
- **Completers** – those who answered all relevant questions based on branching logic

Only Partial Responders and Completers were included in the final analysis.

1.5 Summary of Respondent Categories

The table below shows the distribution of all respondents by category, including those excluded from analysis (Non-Respondents):

Table 1: Summary of Respondent Classifications

Group	Count	Percentage
Completer	62	76.5
Non-Respondent (Viewed Description Only)	11	13.6
Non-Respondent (Viewed but No Responses)	1	1.2
Partial Responder	7	8.6

1.6 Survey Responders

The table below summarizes the respondents included in the final analysis (Partial Responders and Completers):

Total Respondents Analyzed: The final number of respondents analyzed includes both Partial Responders and Completers, for a total of **69** respondents. These respondents provided valuable data that was analyzed and reported throughout this report.

Table 2: Summary of Survey Responders (Partial Responders and Completers)

Group	Count	Percentage
Completer	62	89.9
Partial Responder	7	10.1

Summary of Name and Email Provision: Of the total **69** respondents analyzed, **23** provided either their name or email address. This represents **33.3**% of total respondents. Most respondents did not provide personal information, indicating a preference for anonymity.

Considerations Regarding Response Rate

As noted in the main manuscript, the overall response rate was 33.2% (69 of 208 eligible individuals). This relatively low rate introduces the possibility of response bias—particularly if individuals already using or interested in LLMs were more likely to participate. Because the survey was administered only within biostatistics units at two academic medical centers, the results may reflect early adopters and may not fully represent broader trends across all biostatistical settings.

2 Participant Demographics and Organizational Context

This section provides an overview of the demographics and organizational contexts of survey participants. The questions in this section aimed to capture relevant details about participants' institutional affiliation, roles, and experience levels, which provide a comprehensive backdrop for their perspectives on LLMs.

2.1 Professional Role (Q4 & Q5)

Questions 4 and 5 focused on understanding the professional roles of survey participants. Question 4 asked respondents whether they identified as staff, faculty, or another category, while Question 5 gathered more detailed job title information, such as biostatistician, data scientist, or statistical programmer.

The majority of respondents—**81.2**%—identified as staff, with **17.4**% identifying as faculty. These figures reflect the strong representation of professional staff within the survey cohort, consistent with workforce composition in many research settings.

Question 5 provided additional detail: **82.6**% of respondents identified as biostatisticians, followed by smaller proportions identifying as data scientists, statistical programmers, and other roles.

Table 3: Summary of Respondents' Professional Roles (Q4)

Role	Count	Percentage	Cumulative Percentage
Staff	56	81.2	81.2
Faculty	12	17.4	98.6
Other (please specify)	1	1.4	100.0
Skipped	0	0.0	NA

Table 4: Other Responses for Professional Roles (Q4)

Other (please specify)
Intern

Table 5: Summary of Respondents' Professional Titles (Q5)

Title	Count	Percentage	Cumul Percent
Biostatistician	57	82.6	82.6
Other (please specify)	5	7.2	89.9
Data Scientist	3	4.3	94.2
Statistical Programmer	2	2.9	97.1
Bioinformatician	1	1.4	98.6
Informaticist	1	1.4	100.0
Skipped	0	0.0	NA

Table 6: Other Responses for Professional Titles (Q5)

Other (please specify)
Support Staff
Biostatistician/bioinformatician
Biostatistician intern
Associate Director - Biostatistics
Operations

2.2 Experience Level (Q6)

In Question 6, respondents were asked about their years of experience in their current roles. This question aimed to capture the varying levels of expertise among participants, as experience can significantly influence how individuals adopt and utilize LLMs.

The responses showed a well-distributed range of experience levels. **29.4**% had over 11 years of experience, with similar representation across **26.5**% (0–2 years), **20.6**% (3–5 years), and **23.5**% (6–10 years). This diversity ensures that the survey reflects perspectives from both early-career and experienced professionals, providing a comprehensive view of how experience influences LLM adoption in biostatistics.

Table 7: Summary of Respondents' Experience Levels in Their Current Roles (Q6)

Experience Level	Count	Percentage	Cumul Percent
11+ years	20	29.4	29.4
0-2 years	18	26.5	55.9
6-10 years	16	23.5	79.4
3-5 years	14	20.6	100.0
Skipped	1	1.4	NA

2.3 Institutional Affiliation (Q7)

Respondents were asked to indicate their institutional affiliation – three institutional groups were invited to participate in the survey:

Table 8: Summary of Respondents' Institutional Affiliations (Q7)

Institution	Count	Percentage	Cumul Percent
DCRI Biostatistics	29	42.6	42.6
Stanford QSU	25	36.8	79.4
Duke BERD	14	20.6	100.0
Skipped	1	1.4	NA

Table 9: Summary of Organizational Support for LLM Usage (Q8)

Support Type	Count	Percentage	Cumul Percent
Encouragement to use LLMs	34	49.3	49.3
Guidance or guidelines on appropriate LLM use	13	18.8	68.1
Training on how to use LLMs	13	18.8	87.0
None of the above	9	13.0	100.0
Skipped	0	0.0	NA

2.4 Organizational Support for LLMs (Q8)

Question 8 aimed to determine whether respondents' managing organizations had provided any guidance, training, or encouragement for using LLMs. This question was designed to assess the institutional support available to participants in integrating LLMs into their workflows.

Most respondents reported a lack of organizational guidance or training for LLM use. However, among the 69 respondents who answered this question, 34 (49.3%) noted they received encouragement to explore LLMs, while only 13 (18.8%) had access to formal guidelines and 13 (18.8%) received training. Although this question was intended as a multi-select but implemented as single-choice, the results suggest that while formal support remains limited, many respondents still reported encouragement to use LLMs—reflecting early-stage institutional buy-in.

3 LLM Usage - Key Split

The next section distinguishes LLM users from non-users to understand LLM adoption in biostatistical workflows. It explores usage frequency and engagement, offering insights into adoption trends and practical application among professionals.

3.1 LLM Usage Status (Q9): Overview of LLM Users vs. Non-Users

In Question 9, respondents were asked whether they currently use LLMs in their professional workflows. This item served to distinguish users from non-users and establish a baseline for analyzing subsequent patterns.

The results indicated that a majority of participants—**63.8**%—reported using LLMs in their work. This reflects a substantial rate of adoption among professionals in biostatistics and related fields, underscoring the growing role of LLMs in data-driven research and analytics.

Table 10: LLM Usage Status of Respondents (Q9)

LLM Usage Status	Count	Percentage	Cumul Percent
Yes	44	63.8	63.8
No	25	36.2	100.0
Skipped	0	0.0	NA

3.2 Usage Frequency (Q10): Summary of Frequency of LLM Use

Question 10 asked LLM users how frequently they incorporate these tools into their professional workflows. The goal was to understand not just adoption, but also how regularly LLMs are applied in day-to-day tasks.

Responses varied: **46.5**% reported daily use, and **37.2**% reported weekly use. These results indicate that for many, LLMs have become a routine part of their professional toolkit.

A smaller segment—**14**%—reported using LLMs only on a monthly basis, suggesting variability in usage intensity depending on individual workflows and needs.

Table 11: Frequency of LLM Usage Among Respondents (Q10)

Frequency	Count	Percentage	Cumul Percent
Daily	20	46.5	46.5
Weekly	16	37.2	83.7
Monthly	6	14.0	97.7
Rarely	1	2.3	100.0
Skipped	1	2.3	NA

4 Analysis by LLM Users

This section examines how LLM users incorporate these tools into their professional workflows—including the tools used, types of tasks performed, and perceived utility. It offers insight into the integration of LLMs in biostatistical practice, highlighting both capabilities and limitations.

We report which LLMs are most commonly used, how they support tasks related to communication, clinical knowledge, and quantitative analysis, and how users assess their performance. Each subsection presents detailed perspectives on usage patterns and preferences, providing a comprehensive view of LLM adoption in biostatistics.

4.1 LLM Tools Used (Q11)

Question 11 asked which LLMs respondents use in their workflows. The goal was to assess adoption across commonly available tools. Response options included OpenAI ChatGPT, Microsoft Copilot, Google Gemini, Hugging Face models, and an open-ended "Other" category.

Among the **44** LLM users who answered this question, OpenAI ChatGPT was the most widely used (**75**%), followed by Microsoft Copilot (**50**%). Google Gemini (**20.5**%), Hugging Face tools (**9.1**%), and platforms like Perplexity also appeared in the responses, suggesting both broad adoption and targeted experimentation across platforms.

Table 12: Summary of LLM Tools Used in Biostatistical Workflows (Q11)

Tool	Count	Percentage	Percent of Total
OpenAI ChatGPT	33	75.0	75.0
Microsoft Copilot (formerly Bing Chat)	22	50.0	50.0
Google Gemini	9	20.5	20.5
Hugging Face LLMs	4	9.1	9.1
Anthropic Claude	3	6.8	6.8
GitHub Copilot	3	6.8	6.8
Meta LLaMA	3	6.8	6.8
Other (please specify)	1	2.3	2.3

Table 13: Other Responses for LLM Tools Used (Q11)

Other (please specify)
Perplexity

4.2 LLM Usage Categories

4.2.1 Communication and Leadership Tasks (Q12–Q14)

Questions 12 to 14 explored how respondents use LLMs for communication and leadership tasks, including writing, summarizing, and explaining content—core responsibilities for biostatisticians working in collaborative environments. These items captured both task frequency and perceived usefulness.

Among the **44** LLM users who responded, **6** (**13.6**%) reported not using LLMs for these tasks. Among the **38** active users, the most commonly reported tasks were:

- **Editing and improving writing quality** (**76.3**%)

- **Composing emails or other messages** (**71.1**%)
- **Explaining statistical concepts to non-experts** (**47.4**%)

In terms of perceived usefulness, most respondents described LLMs as **Very useful** (**62.2**%). When asked which LLM they used most often for communication tasks, the top response was **OpenAI ChatGPT** (**62.2**%).

Table 14: Summary of LLM Usage for Communication and Leadership Tasks (Q12)

Task	Count	Percentage	Percent of Total
Editing and improving writing quality	29	76.3	65.9
Composing emails or other messages	27	71.1	61.4
Explaining statistical concepts to non-experts	18	47.4	40.9
Drafting meeting agendas or notes	16	42.1	36.4
Summarizing long documents or conversations	16	42.1	36.4
Writing sections of grant proposals or manuscripts	12	31.6	27.3
Brainstorming ideas or outlining projects	11	28.9	25.0
Drafting recommendation letters or letters of support	11	28.9	25.0
Generating content for presentation slides	8	21.1	18.2
Other (please specify)	4	10.5	9.1

Table 15: Other Responses for LLM Usage for Communication and Leadership Tasks (Q12)

Other (please specify)
Help with Coding in R / SAS. Trying to resolve an error in the code.
Art, Summarizing cultural norms and expectations, defining ideas that I know but don't have time to write out, ...
Learn clinical concepts, ask LLM to provide coding tips
Get programming codes, gain statistical knowledge

Table 16: Usefulness of LLMs for Communication and Leadership Tasks (Q13)

Response	Count	Percentage	Cumul Percent
Very useful	23	62.2	62.2
Somewhat useful	14	37.8	100.0
Skipped	7	15.9	NA

4.2.2 Clinical and Domain Knowledge (Q15–Q17)

Questions 15 to 17 explored how respondents use LLMs for clinical and domain knowledge tasks, such as understanding medical terminology, interpreting clinical measurement scales, and staying informed about developments in their field. These items assessed both usage

Table 17: Most Used LLM for Communication and Leadership Tasks (Q14)

LLM	Count	Percentage	Cumul Percent
OpenAI ChatGPT	23	62.2	62.2
Microsoft Copilot (formerly Bing Chat)	13	35.1	97.3
Anthropic Claude	1	2.7	100.0
Skipped	7	15.9	NA

patterns and perceived usefulness.

Among the **44** LLM users who responded, **9** (**20.5**%) reported not using LLMs for these tasks. Among the **35** active users, the most common applications were:

- **Defining or explaining medical terms and concepts** (**68.6**%)
- **Understanding and interpreting clinical measurement scales** (**40**%)
- **Summarizing scientific papers or reports** (**34.3**%)

Perceived usefulness was generally positive: **Very useful** was selected by **44.1**%, and **Somewhat useful** by **55.9**%. The most commonly used LLM for these tasks was **OpenAI ChatGPT** (**61.8**%).

Table 18: Summary of LLM Usage for Clinical and Domain Knowledge (Q15)

Task	Count	Percentage	Percent of Total
Defining or explaining medical terms and concepts	24	68.6	54.5
Understanding and interpreting clinical measurement scales (e.g., PROMIS, PHQ-9)	14	40.0	31.8
Summarizing scientific papers or reports	12	34.3	27.3
Staying informed about new developments in your field	11	31.4	25.0
Identifying relevant research papers or studies	10	28.6	22.7
Interpreting and utilizing clinical guidelines or best practices	10	28.6	22.7
Critically evaluating research methods in papers, grants, or protocols	6	17.1	13.6
Selecting appropriate outcome measures for specific research questions or studies	6	17.1	13.6
Understanding and interpreting data security guidelines or IRB requirements	1	2.9	2.3

Table 19: Usefulness of LLMs for Clinical and Domain Knowledge Tasks (Q16)

Response	Count	Percentage	Cumul Percent
Somewhat useful	19	55.9	55.9

Table 19: Usefulness of LLMs for Clinical and Domain Knowledge Tasks (Q16) *(continued)*

Response	Count	Percentage	Cumul Percent
Very useful	15	44.1	100.0
Skipped	10	22.7	NA

Table 20: Most Used LLM for Clinical and Domain Knowledge Tasks (Q17)

LLM	Count	Percentage	Cumul Percent
OpenAI ChatGPT	21	61.8	61.8
Microsoft Copilot (formerly Bing Chat)	13	38.2	100.0
Skipped	10	22.7	NA

4.2.3 Quantitative Expertise Tasks (Q18–Q20)

Questions 18 to 20 examined how respondents use LLMs for quantitative expertise tasks, such as coding, statistical analysis, and developing analysis plans. These items assessed both usage frequency and perceived usefulness.

Among the **44** LLM users who responded, **4** (**9.1**%) reported not using LLMs for these purposes. Among the **40** active users, the most common applications were:

- **Writing, debugging, or documenting code** (**77.5**%)
- **Explaining statistical methods or concepts** (**57.5**%)
- **Learning new statistical techniques or software** (**52.5**%)

Perceived usefulness was generally high: **Very useful** was selected by **44.7**%, and **Somewhat useful** by **55.3**%. The most widely used LLM for these tasks was **OpenAI ChatGPT** (**60.5**%).

Table 21: Summary of LLM Usage for Quantitative Expertise Tasks (Q18)

Task	Count	Percentage	Percent of Total
Writing, debugging, or documenting code (e.g., R, Python, SAS)	31	77.5	70.5
Explaining statistical methods or concepts	23	57.5	52.3
Learning new statistical techniques or software	21	52.5	47.7
Interpreting the results of statistical analyses	12	30.0	27.3
Automating repetitive data cleaning or analysis tasks	9	22.5	20.5
Creating or reviewing statistical analysis plans (SAPs)	9	22.5	20.5
Generating synthetic data or simulating datasets	9	22.5	20.5

Table 21: Summary of LLM Usage for Quantitative Expertise Tasks (Q18) *(continued)*

Task	Count	Percentage	Percent of Total
Extracting or transforming data from various sources (e.g., PDFs, websites)	8	20.0	18.2
Performing calculations or data transformations (e.g., generating derived variables, recoding variables, creating summary statistics)	7	17.5	15.9

Table 22: Usefulness of LLMs for Quantitative Expertise Tasks (Q19)

Response	Count	Percentage	Cumul Percent
Somewhat useful	21	55.3	55.3
Very useful	17	44.7	100.0
Skipped	6	13.6	NA

Table 23: Most Used LLM for Quantitative Expertise Tasks (Q20)

LLM	Count	Percentage	Cumul Percent
OpenAI ChatGPT	23	60.5	60.5
Microsoft Copilot (formerly Bing Chat)	10	26.3	86.8
GitHub Copilot	2	5.3	92.1
Anthropic Claude	1	2.6	94.7
Google Gemini	1	2.6	97.4
Meta LLaMA	1	2.6	100.0
Skipped	6	13.6	NA

4.3 Experience with Incorrect LLM Answers (Q21)

Question 21 asked whether respondents had encountered incorrect LLM-generated outputs that could have led to serious consequences. The aim was to understand risks associated with using LLMs in high-stakes biostatistical analysis.

Most respondents reported encountering such errors, highlighting a substantial risk in relying on LLMs for complex tasks. These issues could have resulted in significant downstream effects if not identified, underscoring the importance of verification and oversight in biostatistical workflows involving LLMs.

Table 24: Experience with Incorrect LLM Answers (Q21)

Response	Count	Percentage	Cumul Percent
Yes	29	70.7	70.7
No	12	29.3	100.0
Skipped	3	6.8	NA

4.4 Analysis of LLM Mistakes (Q22)

Q22 Prompt:
Please describe one such situation and the potential impact it could have had on your work if it had gone unnoticed.

This open-ended question asked respondents to elaborate on real-world instances where LLMs produced errors with potentially serious consequences.

4.4.1 Findings from Manual Thematic Analysis

Thematic coding was conducted as described earlier in the appendix. The table below summarizes the primary categories of LLM-related errors identified in responses to Question 22.

Table 25: Primary Categories of LLM Mistakes Identified Through Manual Analysis

Theme	Description	Example Response
**Incorrect Code Generation**	LLMs often generated non-existent functions or incorrectly mixed existing ones. Respondents reported frequent errors in R and SAS code generation, particularly with statistical functions.	Chat GPT has written incorrect R code... It invents functions that don't exist and mixes functions that do exist.
**Statistical Misinterpretation**	Instances where LLMs misinterpreted statistical results, such as reversing the interpretation of odds ratios or providing incorrect statistical conclusions.	...while interpreting the odds ratio, it treated exposure as outcome and vice versa.
**Content Fabrication**	Cases where LLMs generated fictional content or hallucinated non-existent functions, particularly problematic in formal documentation.	...added content to meeting minutes that was not discussed
**Inappropriate Style or Tone**	Responses that failed to match the required professional tone or technical precision, especially in formal communication.	Copilot does not do a good job at drafting emails. It's tone is robotic...

4.4.1.1 Word Frequency Analysis

This analysis counts and ranks the most frequently used terms in respondents' descriptions of

LLM mistakes. It complements the manual thematic coding by highlighting the most commonly discussed issues across free-text entries.

Table 26: Top 10 Most Frequently Occurring Terms in LLM Mistake Analysis

Term	Frequency
copilot	2.9%
code	2.6%
data	2.3%
analysis	1.3%
asked	1.3%
chatgpt	1.3%
incorrect	1.3%
papers	1.3%
sas	1.3%
used	1.3%

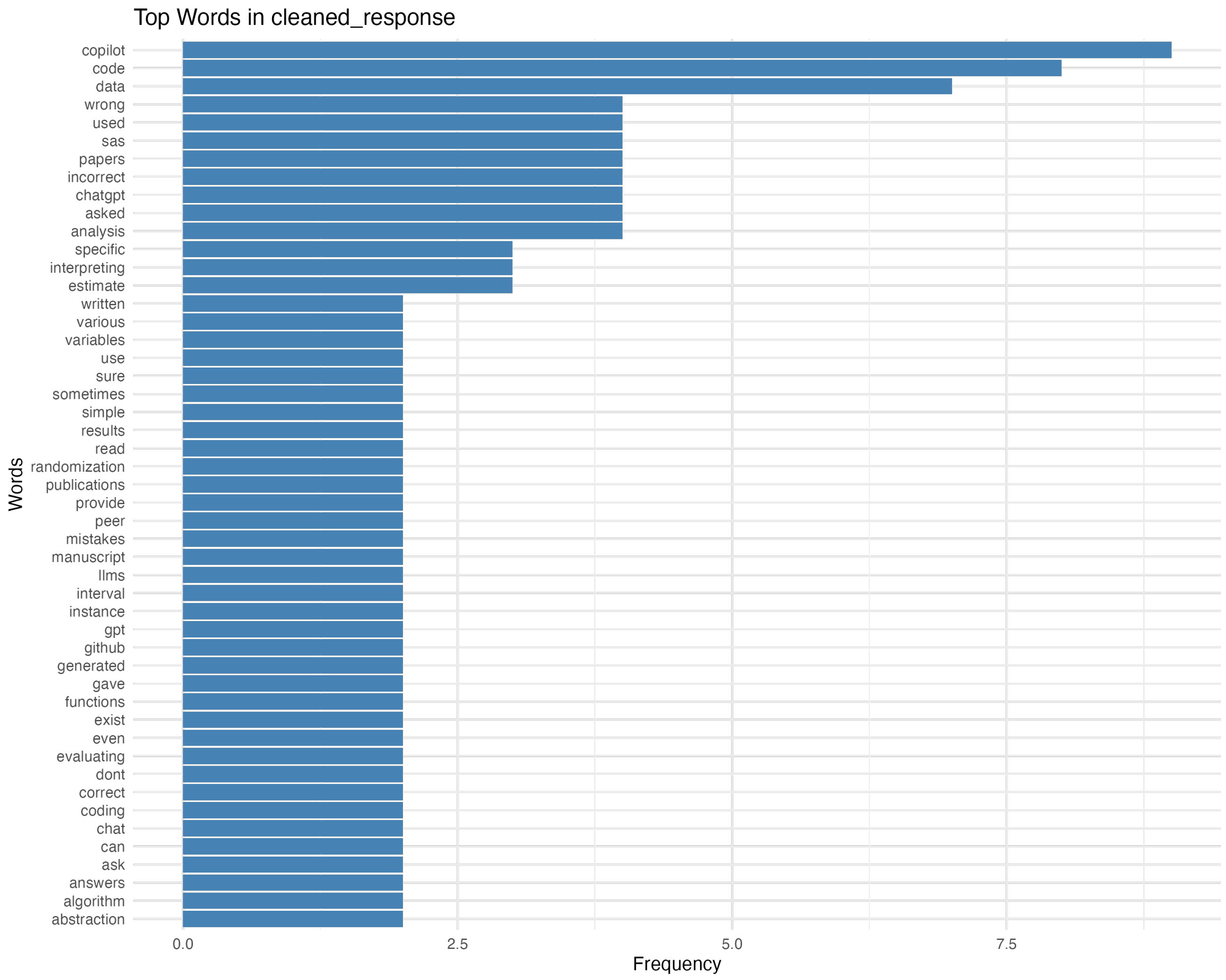

Figure 1: Word Frequency - Q22 Mistakes Descriptions

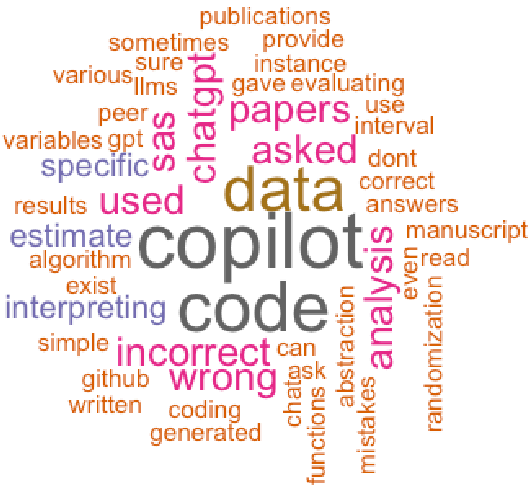

Figure 2: Word Cloud - Q22 Mistakes Descriptions

4.5 Thematic Analysis of How Mistakes Were Recognized (Q23)

Q23 Prompt:

How did you recognize that the LLM's answer was incorrect in this instance?

This open-ended question asked respondents to reflect on the moment they realized an LLM-generated output was flawed. The goal was to surface the strategies professionals use to validate or challenge model outputs in practice.

4.5.1 Findings from Manual Thematic Analysis

Table 27: Thematic Analysis of How Mistakes Were Recognized (Q23)

Theme	Description	Example Response
**Expertise and Prior Knowledge**	Many respondents relied on their domain expertise or prior knowledge to catch mistakes. They compared outputs to known results or recognized inconsistencies due to their familiarity with the subject matter.	I had prior experience with this type of analysis and was aware of the general direction of it.
**Verifying Outputs Through External Means**	Some respondents described web searches and other manual verification methods, such as reading reference papers or checking against human-generated data. This aligns well with respondents' reliance on testing and ground-truthing to verify the outputs of the LLMs.	If I am unsure of an answer, I usually do a web search or check some sources to see if it aligns with what the LLM gave me.
**Testing and Debugging**	Respondents often tested the LLM output through trial and error by implementing the code or checking against their own models. This helped them recognize inconsistencies, failures, or anomalies in the results.	I carefully checked the output on my test data and noticed anomalies. Based on those anomalies, I went back to the code.
**Manual Inspection of LLM Output**	A few respondents mentioned that they carefully reviewed the LLM-generated output, either by inspecting it step-by-step or manually comparing it to known models.	I always do one model manually before making the macro. My manual model did not match the table made with copilot code.

4.5.1.1 Word Frequency Analysis

This analysis counts and ranks the most frequently used terms in responses to Question 23, offering an additional lens on how participants recognized LLM-related mistakes. It complements the manual thematic analysis by highlighting commonly cited indicators of error.

Table 28: Top 10 Most Frequently Occurring Terms in Recognition of Mistakes Analysis

Term	Frequency
code	2.4%
always	1.5%
asking	1.5%
aware	1.5%
checked	1.5%
copilot	1.5%
know	1.5%
output	1.5%
r	1.5%

Term	Frequency
anomalies	1.0%

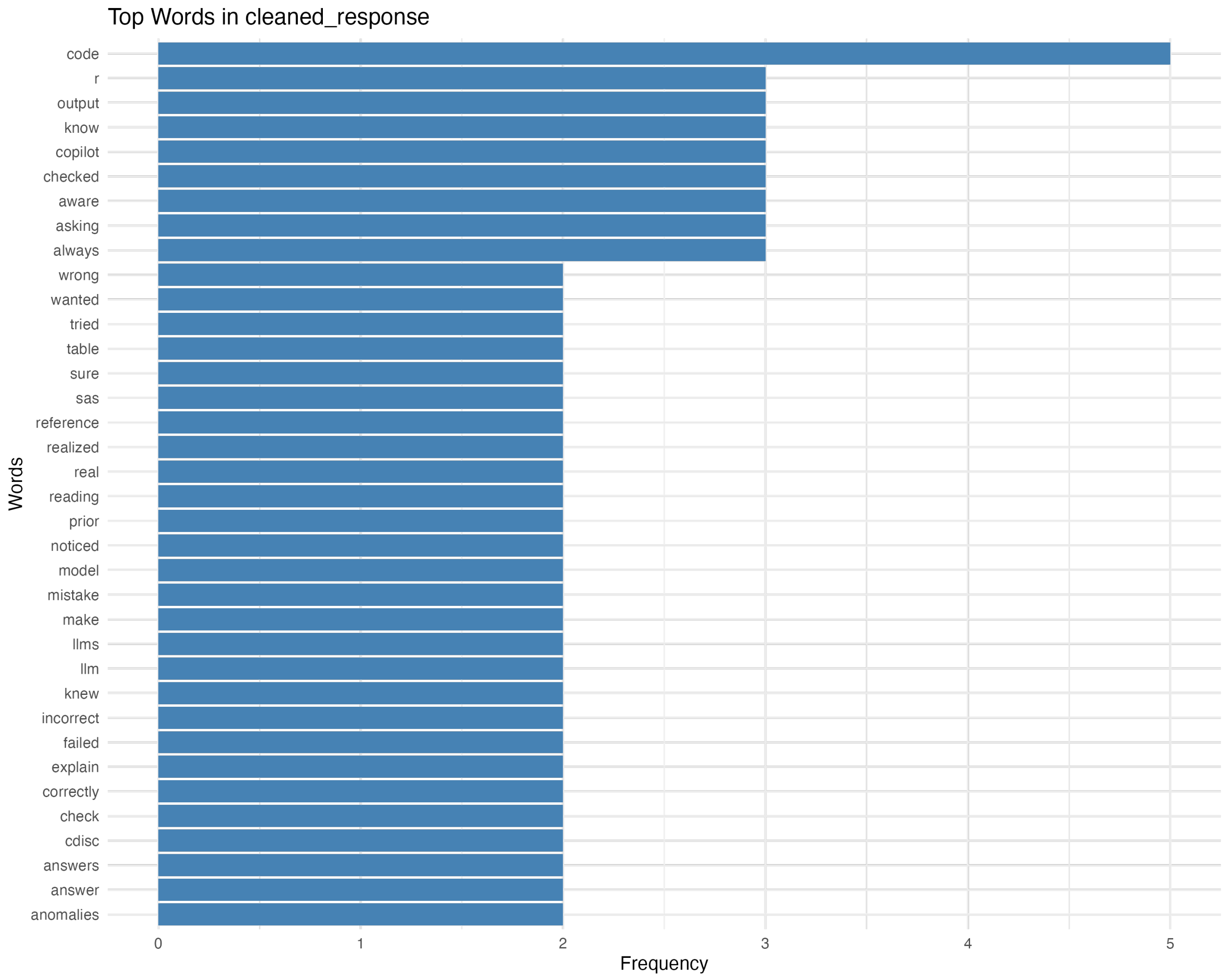

Figure 3: Word Frequency - Q23 Recognition of Mistakes

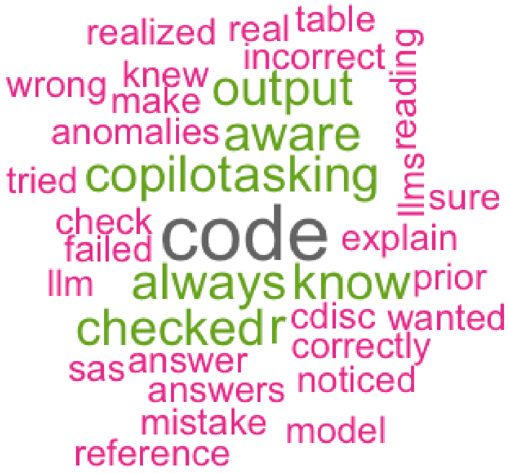

Figure 4: Word Cloud - Q23 Recognition of Mistakes

4.6 Thematic Analysis of Effective Prompts (Q24-Q27)

Q24–Q27 Prompt Set:

Thinking about your use of LLMs across the three key areas we've focused on— communication and leadership, clinical and domain knowledge, and quantitative expertise—please share up to three prompts that you have found particularly effective in your work.

Participants were invited to share up to three free-text prompts that they had used successfully with LLMs. These prompts were intended to surface real-world examples of effective interaction strategies across varied biostatistical workflows.

4.6.1 Findings from Manual Thematic Analysis

Table 29: Thematic Analysis of Effective Prompts Used for LLMs (Q25-Q27)

Theme	Description	Example Response
**Formal Writing Tasks (Emails, Grant Proposals)**	Respondents frequently used LLMs to improve the tone and professionalism of emails, revise research proposals, and edit formal communications.	Please improve/rewrite this email for clarity and conciseness.
**Coding Assistance (R, SAS, Statistical Analysis)**	Many users requested help with coding, including statistical analysis and formatting code for R, SAS, or other programming languages. LLMs were used to troubleshoot, write, or rephrase code snippets for specific tasks.	Help me debug this R code for my analysis of survival data.
**Professional Rephrasing (Clarity, Conciseness)**	Respondents asked the LLMs to rewrite paragraphs or sentences for clarity, conciseness, and professionalism, often for specific audiences such as grant reviewers or collaborators.	Rewrite this sentence for better readability for my collaborator.
**Assuming Expertise/Knowledge Role**	Many prompts included asking the LLM to assume the role of an expert in a particular field (e.g., grant writer, statistician) and assist in providing technical or domain-specific knowledge.	Provide statistical guidance as an expert for a multilevel analysis.
**Scientific References and Resource Assistance**	Some prompts asked LLMs to search for scientific references, peer-reviewed articles, or literature on specific topics.	Find recent peer-reviewed articles on the impact of machine learning in biostatistics.

4.6.1.1 Word Frequency Analysis

This technique counts and ranks the frequency of terms in the responses, helping identify the most commonly discussed themes.

Table 30: Top 10 Most Frequently Occurring Terms in Combined Prompts

Term	Frequency
code	1.7%
include	1.7%
can	1.2%
email	1.2%
use	1.2%
using	1.2%
paragraph	1.1%
provide	1.1%

Term	Frequency
variable	1.1%
grant	0.9%

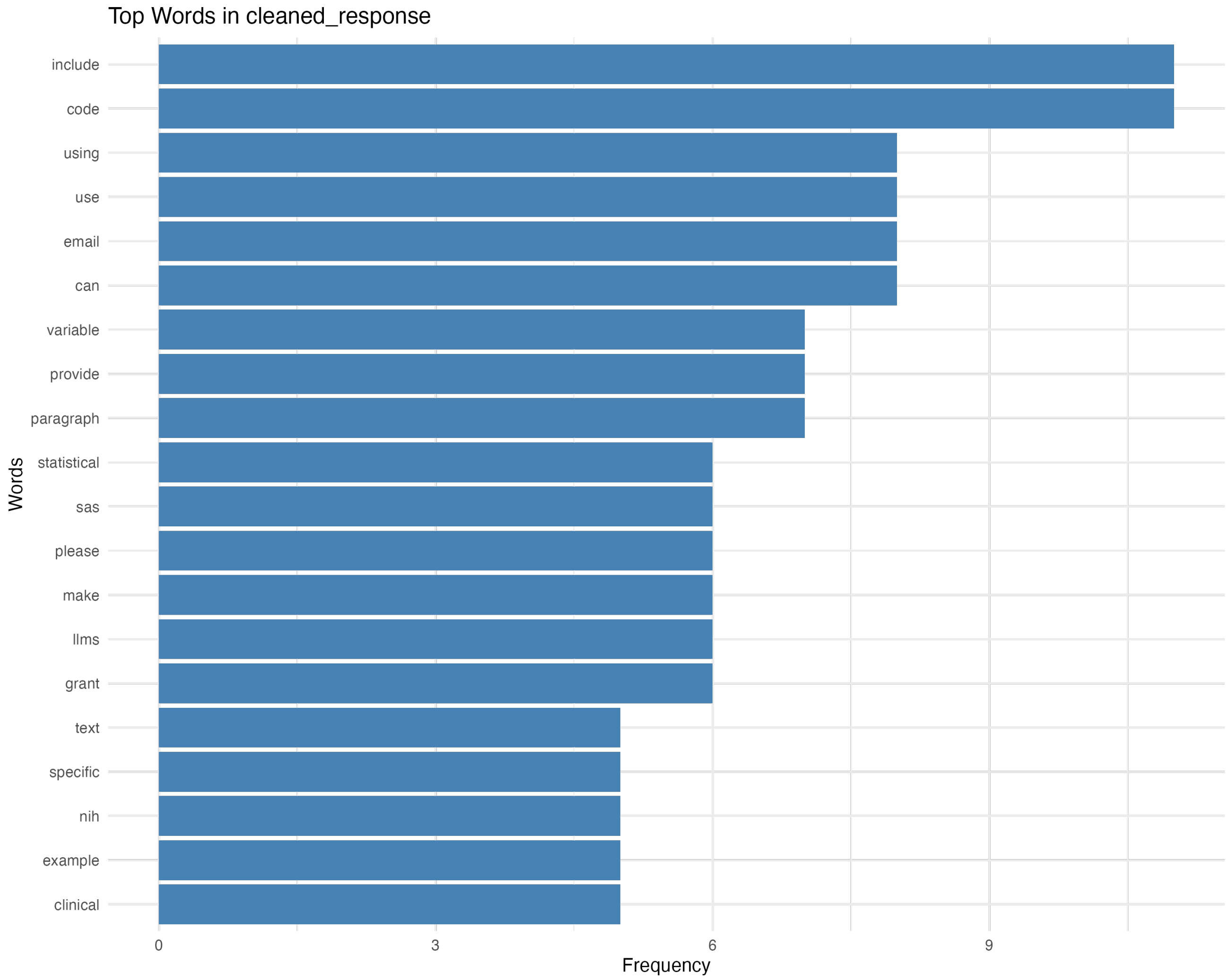

Figure 5: Word Frequency - Combined Prompts

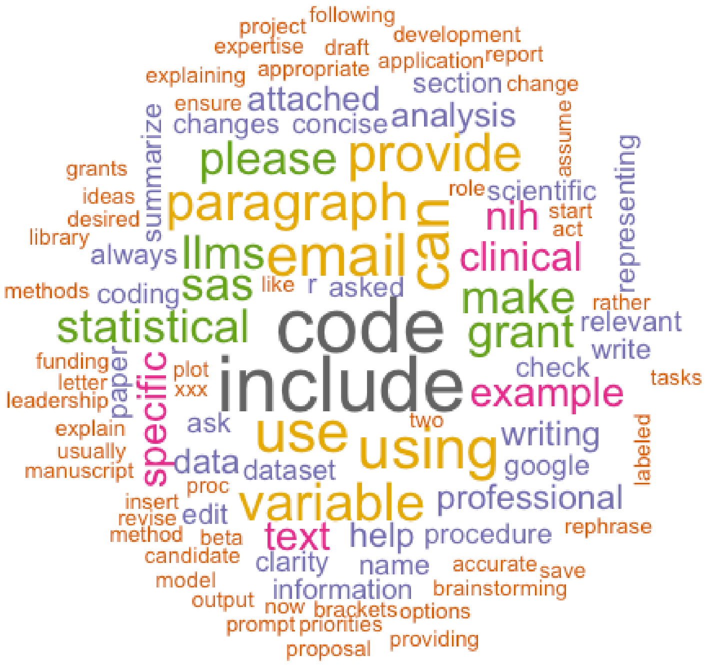

Figure 6: Word Cloud - Combined Prompts

5 Analysis by Non-LLM Users

This section presents insights from respondents who indicated they do not currently use LLMs in their biostatistical workflows. The goal is to better understand reasons for non-adoption, conditions that might encourage future use, and relevant training needs. These insights may help inform more effective implementation strategies across the biostatistical community.

5.1 Reasons for Not Using LLMs (Q28)

Question 28 asked non-users to identify reasons for not using LLMs. Among the **25** respondents, the most frequently cited barriers included:

- **Lack of time to learn how to use LLMs effectively** (**80**%)
- **Concerns about accuracy or reliability** (**44**%)
- **Limited perceived utility for their specific tasks** (**36**%)
- **Insufficient access to training or resources** (**36**%)
- **Data privacy and security concerns** (**20**%)

A few participants also mentioned ethical considerations, environmental impact, or satisfaction with existing tools and processes. These responses highlight key barriers that may need to be addressed to support broader adoption of LLMs in biostatistical settings.

Table 31: Reasons for Not Using LLMs in Work (Q28)

Reason	Count	Percentage	Percent of Total
I haven't had the time to learn how to use them effectively.	20	80	80
I have concerns about the accuracy or reliability of the outputs.	11	44	44
I don't believe they would be useful for the specific tasks I do in my workflow.	9	36	36
I lack access to adequate training or resources on how to use them.	9	36	36
I have concerns about data privacy or security when using LLMs.	5	20	20
I have ethical concerns about the use of LLMs in my field.	1	4	4
Other (please specify)	5	20	20

Table 32: Other Responses for Reasons for Not Using LLMs in Work (Q28)

Other (please specify)
I don't understand what benefit they are expected to provide. I have not seen a clear vision on their benefit to our work.
They take me more time right now because most of the results returned are poor quality and need a lot of work to correct.
Existing methods are working well for my current analyses
I have concerns about environmental impact
Easier to think and work myself than correct the LLM's output

5.2 Incentives for Future Use (Q29)

Question 29 asked non-users what might encourage them to adopt LLMs in their work. Among the **25** respondents, the most frequently selected incentives were:

- **More training or resources specifically tailored to my field** (**80**%)
- **Successful case studies or examples from my colleagues** (**56**%)
- **More evidence of their accuracy and reliability** (**48**%)
- **Clearer guidelines or ethical frameworks for LLM use** (**40**%)
- **Easier-to-use interfaces or platforms** (**40**%)

These findings suggest that providing targeted support and real-world evidence, alongside clearer guidance and accessible tools, could help drive broader adoption of LLMs in biostatistical settings.

Table 33: Incentives for Future Use of LLMs in Work (Q29)

Incentive	Count	Percentage	Percent of Total
More training or resources specifically tailored to my field	20	80	80
Successful case studies or examples from my colleagues	14	56	56
More evidence of their accuracy and reliability	12	48	48
Clearer guidelines or ethical frameworks for LLM use	10	40	40
Easier-to-use interfaces or platforms	10	40	40
Access to a work-approved LLM platform or tool	7	28	28
Improved data privacy and security features	4	16	16
Other (please specify)	1	4	4

Table 34: Other Responses for Incentives for Future Use of LLMs in Work (Q29)

Other (please specify)
Evidence they add efficiency to my workflow

6 Common Questions (Asked to All Respondents)

This section covers questions for all respondents, exploring the needs, challenges, and observations regarding LLM use in biostatistics. Including both users and non-users provides a comprehensive view of expectations and concerns affecting LLM adoption.

6.1 Training Needs for LLMs (Q30)

Question 30 asked respondents about the types of training or resources they would find most helpful for using LLMs in their work. Responses included both users and non-users, highlighting shared gaps in support and knowledge.

Among the **58** respondents, the most frequently selected needs were:

- **Case studies or best practice examples of LLM use in my field** (**75.9**%)
- **Interactive tutorials or guided practice with real-world examples of using LLMs** (**69**%)
- **Structured training sessions or courses (workshops, seminars, webinars, online courses)** (**63.8**%)
- **Comprehensive documentation and user guides for LLMs** (**55.2**%)
- **Mentoring or coaching from experienced LLM users** (**44.8**%)

These findings highlight a preference for practical, hands-on learning opportunities—such as tutorials, case studies, and mentoring—to build confidence with LLM tools and improve productivity in biostatistical workflows.

Table 35: Training Needs for LLMs (Q30) - Summary for All Respondents

Training Type	Count	Percentage	Percent of Total
Case studies or best practice examples of LLM use in my field	44	75.9	63.8
Interactive tutorials or guided practice with real-world examples of using LLMs	40	69.0	58.0
Structured training sessions or courses (workshops, seminars, webinars, online courses)	37	63.8	53.6
Comprehensive documentation and user guides for LLMs	32	55.2	46.4
Mentoring or coaching from experienced LLM users	26	44.8	37.7
Access to online forums or discussion groups focused on LLMs	17	29.3	24.6
Other (please specify)	1	1.7	1.4

Table 36: Other Responses for Training Needs for LLMs (Q30)

Other (please specify)
Time to train

6.2 Analysis of General Observations on LLM Use (Q31)

Q31: Do you have any other thoughts or observations about the use of LLMs in biostatistical workflows that you'd like to share? [Text entry field]

6.2.1 Findings from Manual Thematic Analysis

Table 37: Thematic Analysis of General Observations on Using LLMs in Biostatistical Workflows (Q31)

Theme	Description	Example Response
**Productivity Enhancement**	Many respondents noted significant time savings and increased efficiency in tasks such as coding, writing, and literature review.	LLMs have significantly reduced the time I spend on routine coding tasks and initial draft writing.
**Reliability Concerns**	Concerns were raised about the accuracy of LLM outputs, especially for complex statistical concepts or specialized biostatistical tasks.	While helpful for general tasks, I've found LLMs can sometimes provide incorrect statistical interpretations that require careful checking.
**Need for Critical Oversight**	Respondents emphasized the importance of human verification and the need to maintain a critical perspective when using LLM-generated content.	It's crucial to verify all LLM outputs. They're a helpful starting point, but not a replacement for expert knowledge.
**Domain-Specific Limitations**	Several users pointed out that LLMs sometimes lack in-depth understanding of specific biostatistical methods or domain-specific nuances.	LLMs struggle with newer or highly specialized statistical methods in our field. They often provide generic answers that aren't applicable to our specific research context.
**Future Potential**	Despite challenges, some respondents expressed optimism about the future potential of LLMs in biostatistics, particularly with further refinement and specialized training.	As LLMs continue to improve and potentially receive specialized training in biostatistics, I believe they will become invaluable tools in our workflow.

6.2.1.1 Word Frequency Analysis

This technique counts and ranks the frequency of terms in the responses, helping identify the most commonly discussed themes.

Table 38: Top 10 Most Frequently Occurring Terms in General Observations

Term	Frequency
sas	2.7%
llms	2.2%
can	1.9%
chatgpt	1.6%

Term	Frequency
code	1.6%
copilot	1.4%
good	1.4%
use	1.4%
ask	1.1%
know	1.1%

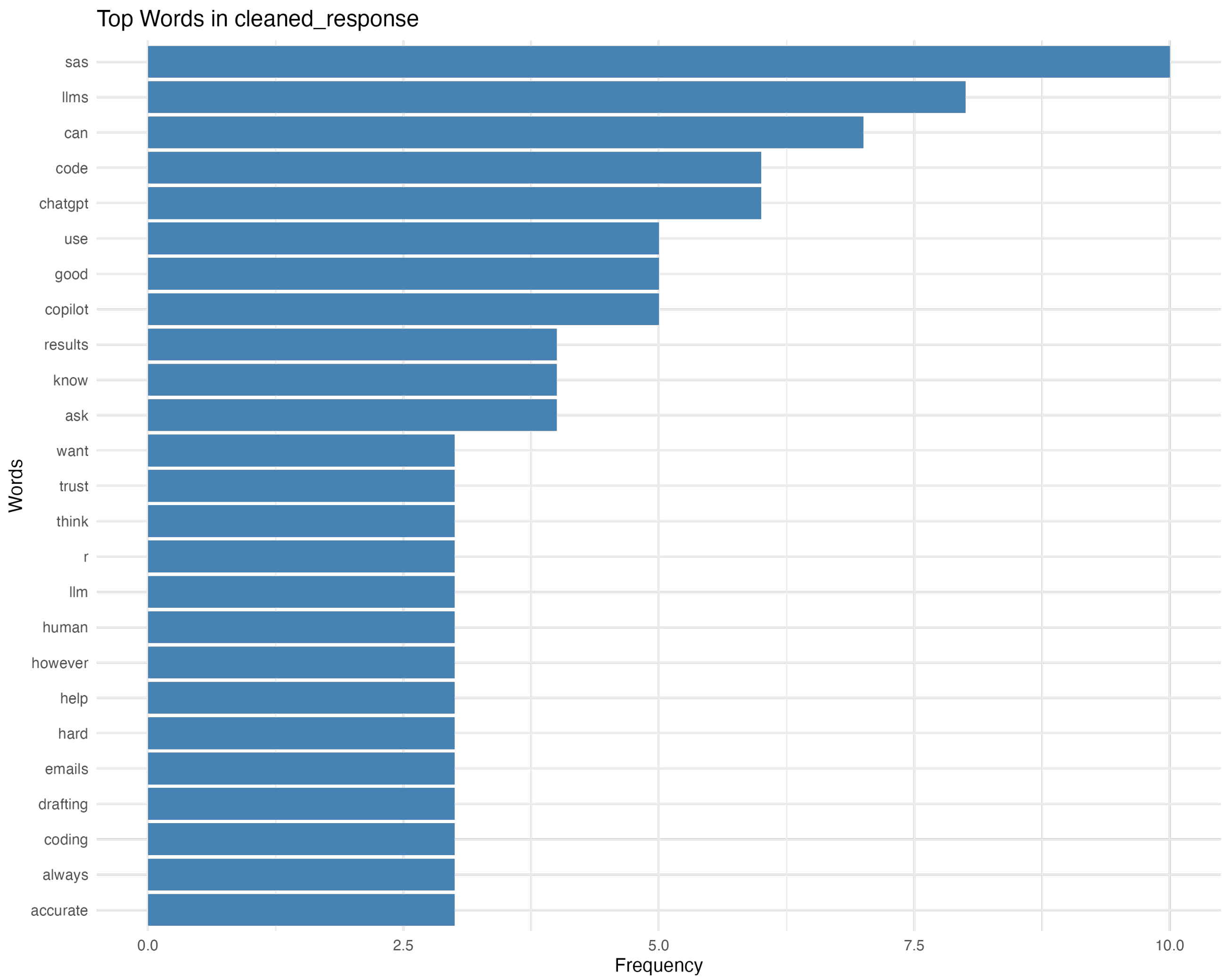

Figure 7: Word Frequency - Q31 General Observations

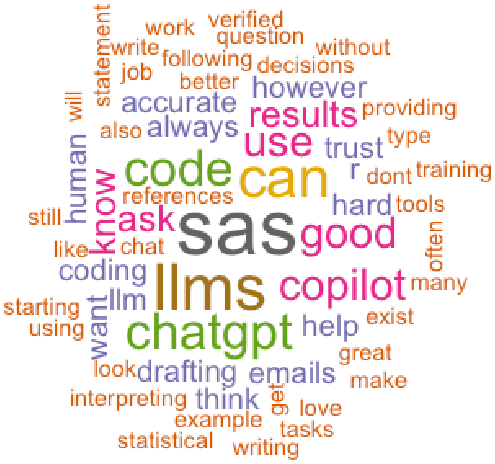

Figure 8: Word Cloud - Q31 General Observations

1. Pomann GM, Boulware LE, Cayetano SM, Desai M, Enders FT, Gallis JA, et al. Methods for training collaborative biostatisticians. Journal of Clinical and Translational Science. 2021;5(1):e26.

2. Slade E, Brearley AM, Coles A, Hayat MJ, Kulkarni PM, Nowacki AS, et al. Essential team science skills for biostatisticians on collaborative research teams. Journal of Clinical and Translational Science. 2023;7(1):e243.

3. Qualtrics XM. Qualtrics survey software [Internet]. Accessed May 4, 2025. Available from: https://www.qualtrics.com/

4. OpenAI. ChatGPT 4o teams edition [Internet]. Accessed May 4, 2025. Available from: https://www.openai.com/chatgpt

5. Anthropic. Claude 3.5 sonnet professional edition [Internet]. Accessed May 4, 2025. Available from: https://www.anthropic.com/claude

6. R Core Team. R: A language and environment for statistical computing [Internet]. Vienna, Austria: R Foundation for Statistical Computing; Accessed May 4, 2025. Available from: https://www.R-project.org/

7. RStudio Team. RStudio: Integrated development environment for r [Internet]. Posit; Accessed May 4, 2025. Available from: http://www.rstudio.com/

8. Quarto Development Team. Quarto: Open-source scientific and technical publishing system [Internet]. Quarto Project; Accessed May 4, 2025. Available from: https://quarto.org/

9. Microsoft. Microsoft copilot [enterprise edition] [Internet]. Accessed May 4, 2025. Available from: https://www.microsoft.com/copilot

10. OpenAI. ChatGPT O1 preview teams edition [Internet]. Accessed May 4, 2025. Available from: https://www.openai.com/chatgpt
